# Combinations of long terminal repeat and tax protein substitutions influence bovine leukemia virus transmission through altered viral productivity

**DOI:** 10.1128/spectrum.00869-26

**Published:** 2026-07-27

**Authors:** Hironobu Murakami, Reiichiro Sato, Jumpei Uchiyama, Kazuyuki Sogawa, Hiroho Ishida, Makoto Nagai

**Affiliations:** 1 School of Veterinary Medicine, Azabu University 47710, Sagamihara, Kanagawa, Japan; 2 Center for Human and Animal Symbiosis Science, Azabu University 47710, Sagamihara, Kanagawa, Japan; 3 Graduate School of Medicine and Veterinary Medicine, University of Miyazaki 12952, Miyazaki, Japan; 4 Department of Infectious Diseases, Graduate School of Medicine Dentistry and Pharmaceutical Sciences, Okayama University 12997, Okayama, Japan; 5 School of Life and Environmental Science, Azabu University 47710, Sagamihara, Kanagawa, Japan; Penn State College of Medicine, Hershey, Pennsylvania, USA; Nagasaki University, Nagasaki, Japan

**Keywords:** bovine leukemia virus, substitution, transmission, haplotype, molecular clone, proviral load, reverse genetics

## Abstract

Bovine leukemia virus (BLV) infection imposes a substantial economic burden on the global cattle industry. Although proviral load (PVL) in blood influences transmission, the effects of viral genetic variations, including the high viral production-associated long terminal repeat (LTR) substitution at nucleotide position 175 (LTR175), on herd-level dynamics remain unclear. This study investigated the effects of specific substitutions on the regulation of viral productivity and transmission. Longitudinal monitoring of a single farm (herd size: 50–68 cows; 438 samples collected) over a 7-year period revealed that viral strains carrying cytosine at LTR175 (LTR175C) significantly increased in frequency, becoming dominant over wild-type strains carrying thymidine. In contrast, amino acid substitutions in the Tax protein at positions 69 (Tax69) and 73 (Tax73) modified viral transactivation independent of LTR175. Structural modeling via AlphaFold2 predicted that Tax69 and Tax73 mutations alter protein properties, suggesting modulation of transactivation activity. Consistent with this prediction, *in vitro* assays confirmed that the substitutions modified viral production by regulating transactivation independent of LTR175. Moreover, high viral-productivity haplotypes containing LTR175C and Tax73Q (glutamine at amino acid residue 73) exhibited a transmission advantage within the herd, even when host PVL levels were not significantly elevated. These findings suggest that cellular-level viral productivity is one of the key determinants of transmission fitness. Overall, this study revealed a regulatory interaction between the LTR and Tax protein that optimizes viral spread and highlights the need for control strategies that account for viral genetic substitutions.

**IMPORTANCE:**

Controlling the spread of bovine leukemia virus (BLV) remains a major challenge for the livestock industry. This study identifies viral genetic mechanisms that enable certain strains to spread more efficiently than others. By integrating *in silico* analyses, *in vitro* experiments, and a 7-year field study on a working farm, we showed that three mutations in viral regulatory regions, i.e., in the long terminal repeat and Tax protein, strongly influence viral success. These substitutions modify viral protein properties and promoter activity, increasing production of new virus particles. Our findings suggested that high-viral-productivity genetic profiles enhance transmission between cattle, allowing specific strains to become dominant within herds over time. This research highlights the importance of monitoring key viral genetic markers to identify high-risk strains and improve strategies to limit the spread of BLV.

## INTRODUCTION

Bovine leukemia virus (BLV), a member of the genus *Deltaretrovirus* in the family *Retroviridae*, causes enzootic bovine leukosis (EBL), a fatal lymphoproliferative disease in cattle. Beyond EBL development, BLV infection substantially affects the livestock industry by reducing milk yield, impairing reproductive performance, and shortening host lifespan (1–3). BLV infection remains highly prevalent in many regions worldwide (4). The virus integrates into host genomic DNA in peripheral blood mononuclear cells as a provirus, establishing persistent infection that evades complete elimination by the immune system (5). This persistence results in severe, ongoing economic losses, making BLV control particularly challenging in countries and regions with high seroprevalence.

Because complete viral eradication is currently impractical, limiting BLV spread is essential for reducing transmission. Certain countermeasures, including prohibiting reuse of medical instruments, heating or freezing colostrum from BLV-infected cows, and controlling biting insects, can inhibit viral transmission. Furthermore, proviral load (PVL) in blood serves as a useful indicator for assessing BLV transmission potential in infected cows (6). Cattle with low PVL, indicating lower infectivity to other animals, often possess a specific bovine leukocyte antigen (BoLA)-DRB3 allele, the most highly polymorphic BoLA class II locus in cattle (7). Therefore, effective countermeasures and transmission indicators exist; however, controlling BLV infection remains challenging in many countries and regions. Consequently, further studies aimed specifically at improving BLV control are required.

Previous research has shown that the BLV genome has a relatively low mutation rate (8). The BLV proviral genome contains long terminal repeats (LTRs) at both the 5′ and 3′ termini, as well as *gag-pro-pol*, *env*, and pX regions between these LTRs. The pX region is further divided into two parts: the microRNA-coding region and the gene-coding region (pX gene), which includes *R3*, *G4*, *tax*, and *rex* (9–12). Our previous study demonstrated that limited substitutions in each gene, microRNA, and noncoding regions generated three groups within genotype I and influenced viral productivity (8), indicating that even small sequence changes can alter viral traits.

We previously reported several substitution and deletion mutations associated with viral productivity (8, 13–15). In one study, a cytosine substitution at nucleotide (nt) position 175 of LTR (LTR175C), associated with high-viral-productivity (HVP) *in vitro*, was predicted to confer a higher transmission potential relative to the wild-type (WT) thymidine strain (LTR175T), which shows lower viral productivity (14). These findings suggest that several substitutions influence viral productivity and transmission-associated traits. Therefore, analyzing the relationships between specific substitutions and transmission fitness could help identify viral strains that impair BLV control. However, the temporal dynamics of LTR175C/T strains in infected cattle on farms have not been investigated. To address this critical gap, the present study significantly extends our previous single-mutation findings (14) by transitioning to a combined multilocus haplotype analysis. Importantly, by leveraging a unique 7-year longitudinal field data set, we tracked the *in vivo* herd dynamics and selection fitness of these viral variants for the first time. Furthermore, we identified novel compensatory and regulatory roles of specific mutations (Tax69M and Tax73Q) that modulate viral productivity. Accordingly, this study analyzed substitutions that have major effects on viral traits to evaluate their potential contribution to within-herd spread.

## RESULTS

### Impact of the LTR175 substitution

**Fig 1:**
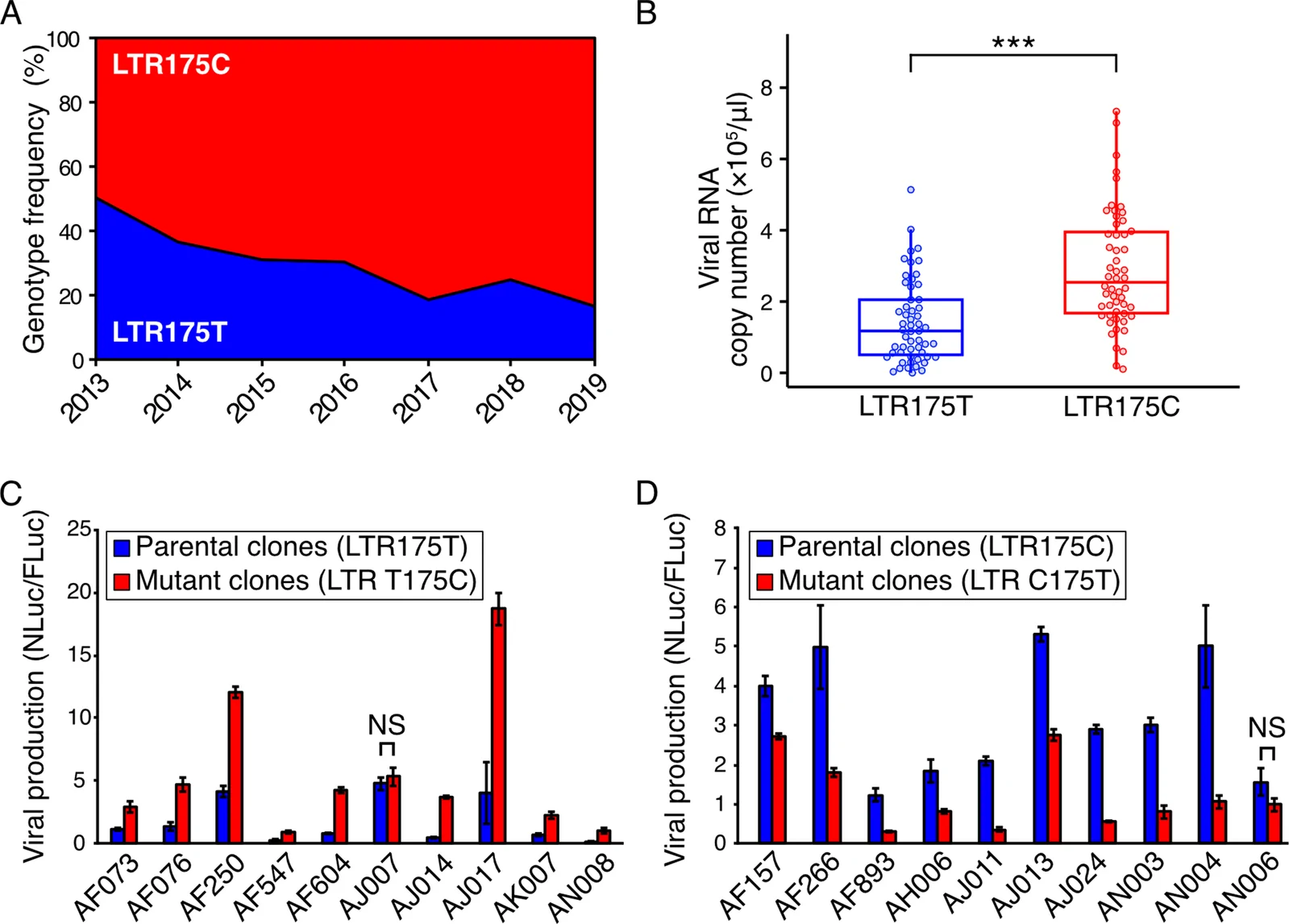
Influence of the LTR175 substitution. (**A**) Dynamics of LTR175 genotype frequencies from 2013 to 2019. Solid blue areas indicate the LTR175T (wild type), whereas solid red areas show the LTR175C (variant). (**B**) Comparison of viral production between LTR175T and LTR175C strains. Dots show the viral copy number of each strain. (***P < 0.001, Mann–Whitney *U* test). (**C**) LTR T175C point mutation in LTR175T parental strains. (**D**) LTR C175T point mutation in LTR175C parental strains. Blue bars indicate parental clones; red bars indicate mutant clones. NS: no significant difference (*P* ≥ 0.05, Student’s *t*-test).

To assess whether HVP strains (LTR175C) become dominant on a single farm (herd size: 50–68 cows) in Tokyo, Japan, infections with wild-type (LTR175T) and variant (LTR175C) strains were monitored during 2013–2019 (Fig. 1A). Infections with LTR175C consistently and significantly increased across the study period (τ = 0.683, *P* = 0.033), whereas those with LTR175T showed no significant trend (τ = −0.350, *P* = 0.282). To exclude confounding effects due to ongoing introductions of BLV-infected cattle from other farms, the introduction frequencies of each strain were examined, and no significant trends were detected (*P* ≥ 0.05). Accordingly, within-farm trends remained unchanged: LTR175C strains showed a significant increase (τ = 0.809, *P* = 0.011), whereas LTR175T strains did not (τ = −0.050, *P* = 0.878). These findings suggest that HVP is associated with increased prevalence. However, whether the functional impact of the LTR175 substitution is affected by other distinct substitutions harbored within each strain remains unknown.

To rule out unique passenger mutations in individual viral backgrounds from confounding these phenotypes, we directly compared viral production across a broad panel comprising 109 distinct BLV isolates. First, we sequenced 29 whole BLV genomes from the aforementioned farm in Tokyo, along with 27 genomes from other prefectures across Japan (Table S1). These 56 strains were cloned into plasmids to construct infectious molecular clones. Additionally, the viral production of 53 previously reported isolates, excluding six deletion strains (AH002, AJ002, AJ020, AJ031, AK004, and AK024) (Table S2), was measured. Determination of viral RNA copy numbers using quantitative PCR revealed that these 109 original clones naturally possessing LTR175C yielded significantly higher viral output as a population compared with those possessing LTR175T, effectively overriding individual genomic background variations (Fig. 1B). Collectively, these findings suggest that HVP contributes to increased viral transmission and prevalence.

To test this hypothesis, reporter infectious molecular clones isolated from BLV-infected cows on the aforementioned farm in Tokyo, as well as previously isolated strains, were constructed, with point mutations subsequently induced at LTR175 using a reverse-genetics approach. Parental and mutation clones, together with pFL-null (an enhancer-free firefly luciferase reporter vector), were transfected into 293T cells to assess viral replication and to compare each parental clone with its corresponding mutant. LTR175 mutations from thymidine to cytosine (LTR T175C) and from cytosine to thymidine (LTR C175T) were each induced in 10 parental clones. LTR T175C mutations significantly increased viral production in most LTR175T strains except AJ007 (Fig. 1C). LTR175C strains showed high viral production, whereas LTR T175C mutations significantly reduced viral production in all strains except AN006 (Fig. 1D). To confirm whether these phenotypic outcomes were consistent in bovine cells, representative clones (AJ017 and AJ013) and the specific variant strains (AJ007 and AN006) were transfected into bovine MAC-T cells. Consistent with the observations in 293T cells, the LTR T175C mutation significantly enhanced viral production in AJ017, whereas the C175T mutation reduced viral production in AJ013. There were no significant changes in viral production in AJ007 and AN006 (Fig. S1). Consequently, only 2 of 20 strains (AN006 and AJ007) showed no significant changes, indicating that the LTR175 substitution influences viral productivity in ~90% of wild-type strains. In contrast, other substitutions modulate viral output independent of LTR175. Notably, strain AF547, which possesses the same LTR175T, Tax69T, and Tax73Q genotype as AJ007, did not exhibit the expected high viral productivity phenotype (Fig. 1C). Similarly, strain AN003, which shares the same LTR175C, Tax69M, and Tax73R genotype as AN006, did not exhibit the LVP phenotype observed in AN006 (Fig. 1D). Together, these observations suggest that while the LTR175 substitution is the primary determinant of viral productivity in most strains, additional genetic variations outside the locus may modulate the phenotype in certain strain backgrounds.

### Influence of *tax* gene nt substitutions on protein structure

**Fig 2:**
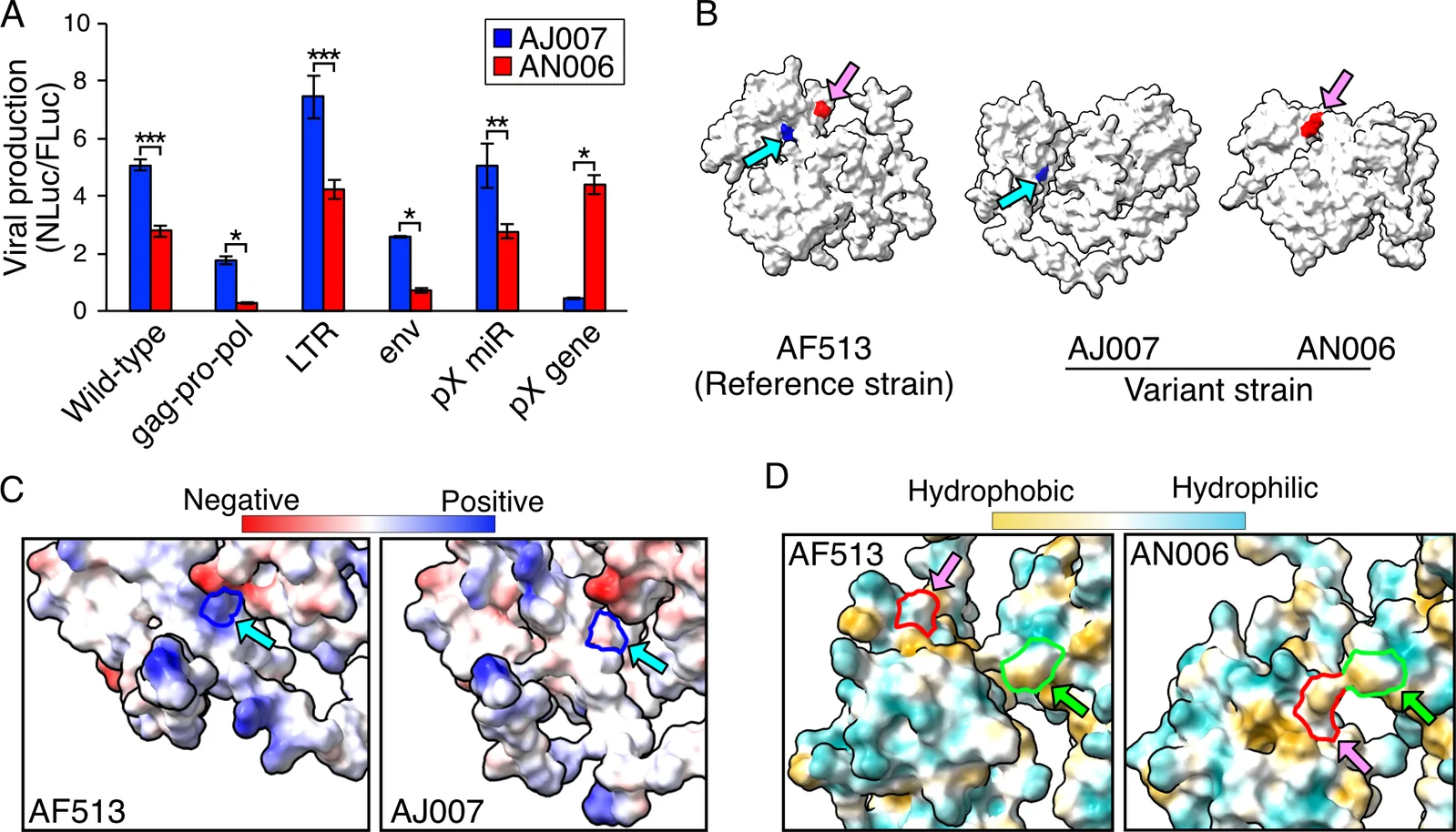
Genome-wide screening of viral productivity and AlphaFold2-predicted surface structures of the Tax protein. (**A**) Comparison of viral production among wild-type, chimeric, and recombinant reporter molecular clones in 293T cells. Blue and red bars indicate reporter clones with AJ007 and AN006 genomic backbones, respectively. The specific genomic regions exchanged (LTR, *gag-pro-pol*, env, pX miR, and pX gene) are indicated on the horizontal axis. Statistical significance was determined using Student’s *t*-test (*P < 0.05; **P < 0.01; ***P < 0.001). (**B**) Close-up global structural comparison of Tax proteins shown as surface representations for the AF513 reference strain, AJ007 variant, and AN006 variant. Magenta and light blue arrows indicate the positions of residues 69 and 73, respectively, corresponding to the two missense mutation sites. (**C**) Close-up electrostatic surface potential models of the AF513 (left) and AJ007 (right) Tax proteins. Light blue arrows indicate the position of residue 73, emphasizing the substantial diminution of the localized positive charge (blue region) resulting from Q73 substitution in AJ007. The color scale indicates negative (red) to positive (blue) electrostatic potentials. (**D**) Hydrophobicity surface models of the AF513 (left) and AN006 (right) Tax proteins. Magenta and green arrows indicate the position of residues 69 and 146, respectively. All surface models in panels B, C, and D are visualized from the same spatial viewing orientation, strictly aligned via structural superposition with the ChimeraX Matchmaker tool.

To identify genetic determinants that modulate viral productivity independently of the LTR175 genotype, we focused on a highly intriguing phenotypic inversion observed between two distinct field isolates: strain AJ007, which harbors LTR175T but unexpectedly exhibits HVP, and strain AN006, which harbors LTR175C but paradoxically exhibits low viral productivity (LVP). Genomic alignment of these two strains revealed 60 nucleotide differences scattered across the LTR, *gag-pro-pol,* AS2, *env*, AS1, *G4, tax*, and *rex* regions (Fig. S2). To investigate at a genome-wide level that genomic regions harbor the non-LTR175 background variations responsible for driving or suppressing total viral production, we performed region-specific recombination using reporter virus-coding molecular clones (pBLVR-AJ007 and -AN006) to construct a panel of chimeric reporter clones (Fig. S3). Following the transfection of 293T cells with these constructs along with pFL-null, viral production and transfection efficiency were evaluated via luciferase assays. Reflecting their baseline characteristics, viral production was significantly higher in the clones carrying AJ007 backbones than those with AN006 backbones; however, this difference was abolished or modified when the pX gene region was recombined (Fig. 2A).

In the pX gene region, 6 nt differences (nt 7,012, 7,475, 7,487, 7,914, 7,989, and 8,016; Fig. S2) distinguished the strains from the consensus sequence. Notably, only the substitutions at nt 7,475 and 7,487 in the *tax* gene produced missense mutations, whereas the remaining four variants were synonymous substitutions. A cytosine-to-thymidine substitution at nt 7,478 resulted in a threonine-to-methionine substitution at Tax residue 69 (Tax69), whereas a guanine-to-adenine substitution at nt 7,487 caused an arginine-to-glutamine substitution at Tax residue 73 (Tax73). Consequently, AJ007 carries threonine and glutamine at Tax residues 69 and 73, respectively (Tax69T/Tax73Q), whereas AN006 carries methionine and arginine at the corresponding positions (Tax69M/Tax73R). Given that Tax upregulates viral transcription via the LTR (16–18), these specific natural variants were hypothesized to influence viral production.

The impact of these missense mutations on protein structure was analyzed using AlphaFold2, comparing the Tax protein across AJ007, AN006, and the reference strain AF513. To ensure a rigorous and direct comparative analysis across all AlphaFold2-predicted models, the spatial viewing orientation of each Tax protein was strictly aligned via structural superposition using the ChimeraX Matchmaker tool. To evaluate the overall molecular architecture, the predicted global fold was initially examined using surface representations, revealing a highly comparable three-dimensional shape across the strains (Fig. 2B). In Fig. 2B, the magenta and light blue arrows indicate the positions of residues 69 and 73, respectively, corresponding to the two missense mutation sites identified in this study. To investigate the structural backbone in detail, structural superposition using the Matchmaker tool was performed with ribbon models (Fig. S4A). Structural superposition via the ChimeraX Matchmaker tool showed strong conservation in the core regions, with pruned root mean square deviations (RMSDs) of 1.29 Å (39 atom pairs) for AJ007 and 1.32 Å (56 atom pairs) for AN006, suggesting global fold preservation. Including all residues revealed larger global RMSDs (16.6 Å for AJ007 and 11.2 Å for AN006), reflecting structural divergence in flexible or peripheral regions (Table S3). Electrostatic surface potential analysis predicted that the Tax73Q variant in AJ007 markedly altered the local electrostatic environment on the protein surface (Fig. 2C; Fig. S4B); the loss of the positively charged arginine (R73) in the reference strain and its replacement with a neutral glutamine (Q73) was modeled to significantly diminish the localized positive surface charge (Fig. 2C). This charge alteration likely contributes to the observed regional structural deviation in the AJ007 variant. In strain AN006, hydrophobicity surface modeling showed that the Tax69M variant introduces a hydrophobic methionine residue (M69) instead of the wild-type threonine (T69) (Fig. S4C). This substitution was predicted to facilitate a novel local hydrophobic contact with residue V146 (indicated by green arrows in Fig. 2D), suggesting a stabilizing effect on surface region interactions and contributing to the increased all-atom RMSD (11.199 Å). The conserved core region likely maintained transcriptional function, explaining the sustained viral productivity of both strains, whereas these predicted peripheral structural changes to Tax may have modulated Tax-mediated transcription and viral production.

### Effects of tax mutations on viral productivity via transactivation

**Fig 3:**
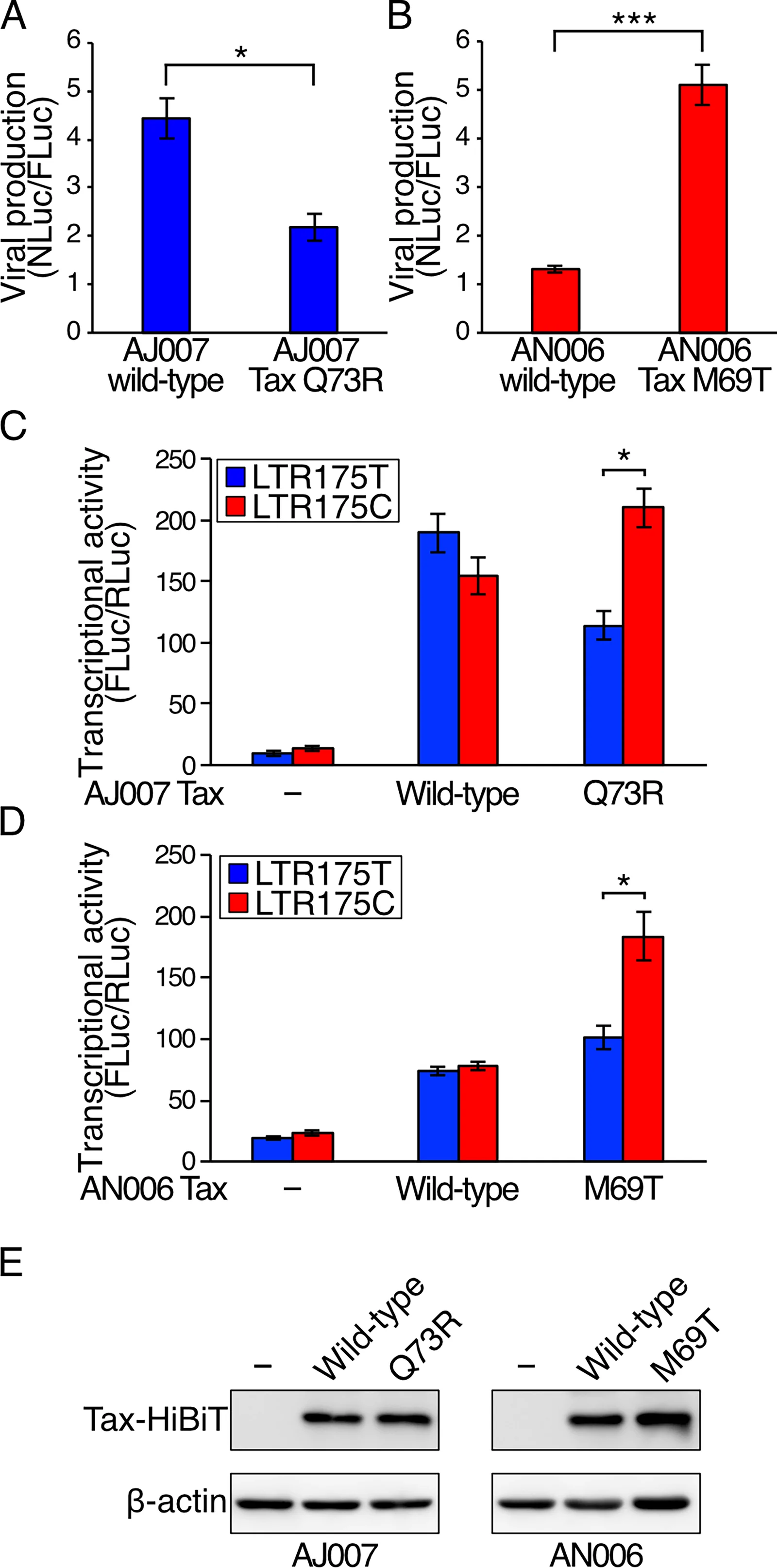
Analysis of Tax mutations. (**A**) Comparison of viral production between wild-type AJ007 and the TaxQ73R mutant. (**B**) Comparison of viral production between wild-type AN006 and the TaxM69T mutant. In panels A and B, blue and red bars indicate AJ007 and AN006 genetic backbones, respectively. (**C, D**) Transactivation activity of wild-type and mutant Tax proteins under the control of LTR175T (blue bars) and LTR175C (red bars) LTR sequences in the AJ007 (**C**) and AN006 (**D**) backgrounds. Samples using an empty vector without Tax transfection served as negative controls (indicated as “–”). Statistical significance was determined using Student’s *t*-test (*P < 0.05; **P < 0.01; ***P < 0.001). NS: no significant difference (*P* ≥ 0.05). (**E**) Western blot analysis of Tax protein expression levels in 293T cells. Cellswere transfected with an empty vector (negative control) or expression vectors encoding wild-type or mutant Tax proteins (AJ007 wild-type, AJ007 Tax Q73R, AN006 wild-type, and AN006 Tax M69T) under the control of the constitutive EF1α promoter.

To assess the effects of both mutations on viral productivity, Q73R and M69T mutations were introduced into the AJ007 and AN006 reporter molecular clones, respectively. Results revealed that AJ007 carrying the Tax Q73R mutation produced significantly less virus compared with the wild-type (Fig. 3A), whereas AN006 carrying the Tax M69T mutation produced significantly more virus relative to the wild-type (Fig. 3B). These results indicate that viral production is regulated by Tax transcriptional activity independent of the LTR175 substitution.

To determine whether viral transactivation regulation is stronger with Tax mutations or the LTR175 substitution, the transcriptional activity of each Tax protein was evaluated given the LTR175T and LTR175C sequences. To strictly distinguish between basal, Tax-independent transcription and Tax-mediated transactivation, a negative control using an empty vector was included (indicated as “–” in Fig. 3C and D). In the absolute absence of Tax, the LTR175T and LTR175C promoters exhibited equivalent baseline activities (*P* ≥ 0.05). Furthermore, when the original wild-type Tax proteins were supplied, there was no significant difference in transcriptional activity regardless of the LTR175 genotype. Specifically, AJ007 Tax wild-type protein sustained robust and equally high transactivation levels under the LTR175T and LTR175C backgrounds (Fig. 3C). Conversely, AN006 Tax WT exhibited markedly low transactivation profiles, showing no preference or elevation under either LTR background (Fig. 3D). Introduction of the specific single-residue mutations resulted in transcriptional activity that was dependent on the substitution at LTR175, resulting in a significantly lower transcriptional output compared with LTR175C (Fig. 3C). In contrast, under the AN006 background, the M69T substitution rescued and enhanced its transactivation capability, driving a significantly higher transcriptional response selectively toward the LTR175C promoter (Fig. 3D).

To elucidate the underlying mechanism behind these distinct transactivation profiles, we next investigated whether these single-residue substitutions affected Tax protein intracellular stability or expression. Western blot analysis of the Tax-HiBiT fusion proteins in 293T cells revealed that Q73R or M69T introduction did not alter Tax protein expression, with all variants sustaining robust, equivalent bands comparable to their respective wild-types (Fig. 3E). This crucial observation rules out the possibility that the altered transactivation was due to fluctuations in Tax levels, conclusively demonstrating that these mutations impact the qualitative transactivation efficiency via structural or interaction-dependent dynamics on the LTR promoter.

To determine whether these mutations are rare, we compared whole genomes of 115 strains shown in Table S1 and S2. The result revealed that the R73Q and T69M mutations occurred in only 2.6% (3/115) and 5.2% (6/115) of the total strains, respectively (Fig. S5). Thus, the combined frequency of both mutations was <10%. This result is consistent with the findings shown in Fig. 1C and D, indicating that the LTR175 substitution is the primary determinant of viral productivity in >90% of the strains examined. However, exceptions to this general trend were observed. Strain AF547, which carries the same combination of substitutions (LTR175T, Tax69T, Tax73Q) as the HVP strain AJ007, did not exhibit the expected high viral productivity (Fig. 1C). Similarly, strain AN003, which possesses the same combination of substitutions (LTR175C, Tax69M, Tax73R) as the LVP strain AN006, did not exhibit the LVP phenotype (Fig. 1D). Whole-genome comparison revealed that these strains (AF547 vs AJ007 and AN003 vs AN006) carried distinct nucleotide substitutions across multiple regions of the viral genome (Fig. S6). Some of these substitutions may affect the viral phenotype, as they include both LTR mutations and missense mutations in coding regions. Specifically, AF547 carries not only an LTR mutation (nt 160) but also missense mutations in p4 (nt 939) and polymerase coding regions (nt 3,605) compared with AJ007 strain (Fig. S6A). Similarly, AN003 strain carries an LTR mutation and a missense mutation in the *G4* gene (nt 7,195) that is absent in AN006 (Fig. S6B). Collectively, these observations indicate that while LTR175 and the two Tax substitutions are key determinants of viral productivity, additional genetic variations outside these loci can modulate the phenotype in certain strain backgrounds.

### Haplotype identification and temporal dynamics

To determine whether specific combinations of the three identified regulatory loci confer a transmission advantage, we genotyped LTR175, Tax69, and Tax73 in field samples and analyzed their dynamics within the farm population over the 7-year study period. Genotyping of blood samples collected from cattle on the abovementioned farm in Tokyo between 2013 and 2019 identified four substitution combinations, defined as haplotypes: T-T-R, C-T-R, T-T-Q, and T-M-R (corresponding to nt or amino acid states at LTR175, Tax69, and Tax73). The T-T-R haplotype was considered the wild-type, based on the consensus sequence derived from the majority-rule alignment of 115 BLV strains (Table S1 and S2; the nucleotide and amino acid frequencies at the three loci are summarized in Fig. S5), with the others representing variants. They were further grouped by viral productivity into HVP haplotypes (C-T-R and T-T-Q), containing LTR175C or Tax73Q associated with high viral production, and LVP haplotypes (T-T-R and T-M-R), including the wild-type and Tax69M variant showing lower productivity.

**Fig 4:**
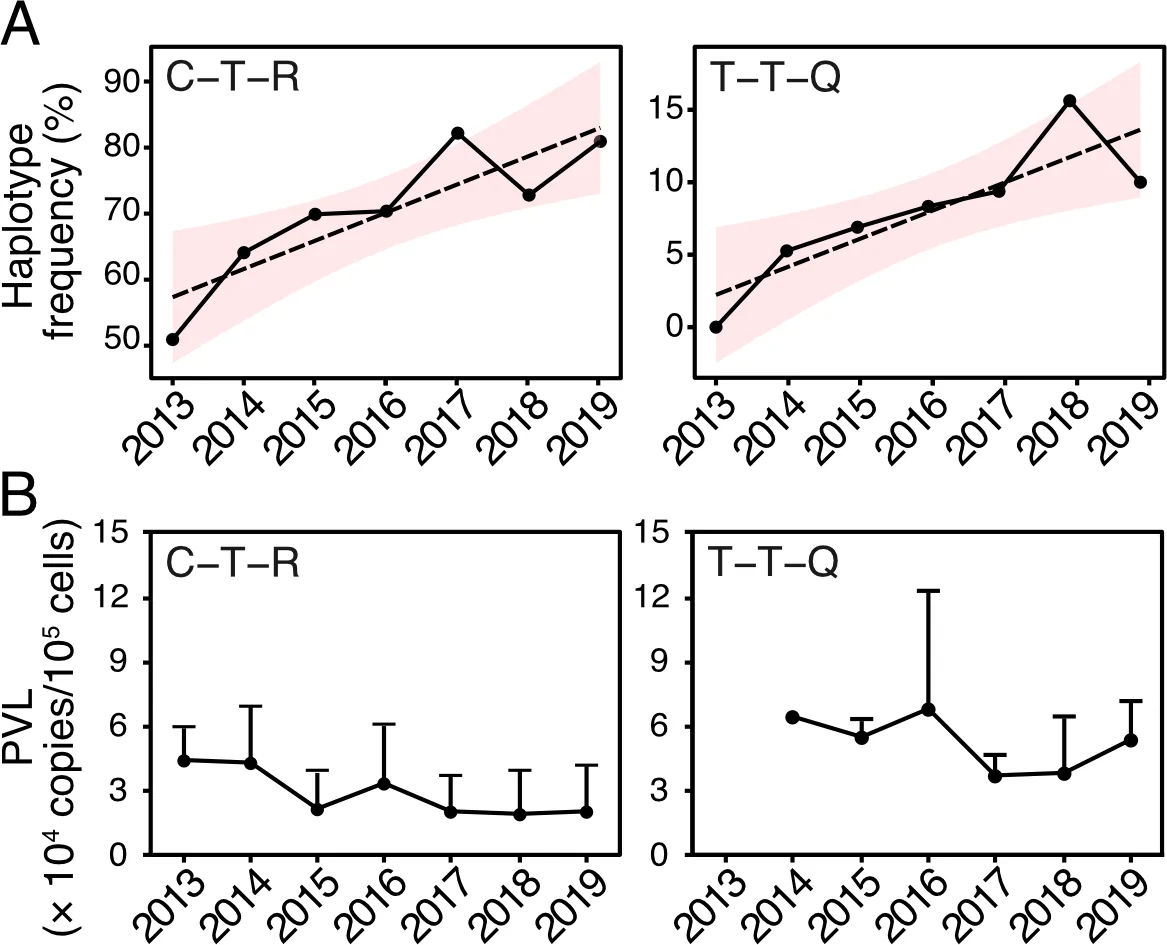
Longitudinal analysis of BLV strain frequencies and PVL in HVP haplotypes. (**A**) Annual relative frequencies of HVP haplotypes. Solid black lines show observed trends; dashed black lines indicate linear regression, with red-shaded areas denoting the 95% confidence intervals of regression. (**B**) PVLs among haplotypes. PVL is plotted for HVP haplotypes as means ± standard deviations for each year.

To determine whether specific strains showed transmission advantages on the farm, annual relative frequencies of the HVP haplotypes—calculated using a standardized unit of one sample per animal per year—were analyzed over the 7-year study period (Fig. 4A), with these haplotypes exhibiting significant increases in relative proportion over time (C-T-R: τ = 0.810, *P* = 0.011; T-T-Q: τ = 0.905, *P* = 0.003). To exclude potential confounding from cattle introduced from other farms, the analysis was repeated after removing animals with an introduction history, focusing on within-farm transmission dynamics using only longitudinal persistent records. Again, significant increases in HVP haplotypes were detected (C-T-R: τ = 0.905, *P* = 0.003; T-T-Q: τ = 0.951, *P* = 0.004). These findings suggest that both HVP haplotypes have a distinct advantage in within-farm horizontal transmission. In contrast, LVP haplotype frequencies did not change significantly on farms over time within the farm population compared with HVP haplotypes (Fig. S7A). Thus, only specific strains appear to exhibit enhanced transmission fitness within the farm population.

To assess whether transmission advantages were associated with PVL, PVL was measured in cattle sampled during 2013–2019 (Fig. 4B and Fig. S7B). After adjusting for years since entry and individual variation using a linear mixed model, estimated geometric mean PVL values were calculated for HVP (C-T-R: 8,045 copies/10⁵ cells; T-T-Q: 24,624 copies/10⁵ cells) and LVP (T-T-R: 10,554 copies/10⁵ cells; T-M-R: 83,662 copies/10⁵ cells) haplotypes. Notably, HVP haplotypes carrying substitutions advantageous for transmission showed numerically lower PVLs compared with the LVP haplotype. However, pairwise comparisons with Tukey adjustment showed no significant differences between haplotypes (*P* ≥ 0.05 for all comparisons; Table S4), indicating that PVL alone does not explain the observed shifts in haplotype dominance. These findings suggest that haplotypes influence transmissibility independent of PVL.

## DISCUSSION

This study showed that specific nt substitutions in the LTR and the *tax* gene of BLV significantly influence viral productivity via transcriptional activity, which may contribute to enhanced transmission fitness within the herd. Longitudinal monitoring over 7 years revealed a marked shift in the viral population, with HVP haplotypes (C-T-R and T-T-Q) showing a consistent, significant increase in frequency. Conversely, LVP haplotypes did not exhibit similar dominance. Importantly, *in silico* analysis, reverse genetics, and reporter assays indicated that the LTR175C substitution and Tax mutations independently altered viral transactivation and production, potentially mediated by predicted structural changes, without altering protein expression levels. Although the LTR175 substitution determines viral productivity in ~90% of surveyed strains, rare Tax mutations (Tax73Q and Tax69M) can override this effect. Notably, this advantage was not associated with higher PVL in individual cows, as no significant PVL differences were detected among haplotypes. Our previous study also showed that cows infected with LTR175C strains did not differ significantly in PVL relative to those infected with LTR175T strains (14). Thus, relatively lower PVL in cattle infected with HVP haplotypes carrying the LTR175C substitution is consistent with prior findings. While our previous work mainly focused on the *in vitro* characterization of the single LTR175C mutation (14), the current study shifts the paradigm to the analysis of combined multilocus haplotypes. Crucially, we provide the first longitudinal *in vivo* evidence that this molecular advantage translates into long-term population-level dominance over a 7-year period, while uncovering novel regulatory roles of Tax69M and Tax73Q that were completely unrecognized in earlier single-locus evaluations.

The Tax protein plays a key role in regulating transcription of the viral genome (10). Previous studies identified several amino acid residues associated with transcriptional activation (19); however, the substitutions detected in the current work occurred at different positions. Notably, the identified substitutions were not located within previously characterized functional domains (20). Moreover, both substitutions regulated viral productivity independent of the LTR175 nt substitution. The LTR175 site lies immediately downstream of the TATA box (21), a critical region where the substitution likely alters the assembly or initiation efficiency of the basal transcription machinery. In contrast, the absence of synergistic or additive effects between the LTR and Tax mutations provides insights into the regulatory constraints of BLV transcription. Specifically, the combination of LTR175C and Tax73Q did not further increase transactivation beyond their individual maxima, nor did the LTR175T and Tax69M combination produce an additional decrease. These findings indicate that coordinated interactions between LTR and Tax sequences influence viral productivity and suggest that haplotype identification is important for estimating transmissibility. Nevertheless, the observation that strains sharing identical LTR and Tax genotypes can exhibit different viral productivity phenotypes (AN003 vs. AN006; AF547 vs AJ007) indicates that additional genetic variations outside these three loci also contribute to viral output. Whole-genome comparisons identified candidate modifiers in these strains, including two missense mutations in the *gag-pro-pol* gene (nt 939 and 3605) in AF547 and a missense mutation in the *G4* gene (nt 7195) in AN003. The mutation at nt 939 is located adjacent to the PPPY motif, which regulates viral particle production, whereas the mutation at nt 3605 lies within the polymerase, which is critical for viral replication (22, 23). Likewise, G4 is known to regulate viral production (13, 15). These findings suggest that viral phenotype is governed by a broader genetic network involving multiple loci.

Previous studies demonstrated that PVL is closely associated with BLV transmissibility (7, 24). However, our results show that PVL is not necessarily higher in HVP haplotypes than in LVP haplotypes. For example, cattle infected with the T-T-Q haplotype showed PVLs associated with elevated transmission risk, whereas other haplotypes did not (< 12,000-20,000 copies/10⁵ cells) (25, 26). This discrepancy suggests a two-step transmission model: first, higher PVL may increase the likelihood that infected cells transfer to a new host; second, once infection occurs, intrinsic viral production capacity determines whether the virus efficiently propagates to uninfected cells. Therefore, HVP haplotypes may overcome transmission bottlenecks even at moderate PVL levels. Indeed, the T-T-Q haplotype, combining relatively high PVL with HVP, exhibited a slightly steeper increasing trend relative to C-T-R (C-T-R vs T-T-Q: τ = 0.905 vs τ = 0.951), suggesting it could become dominant over time. Although BLV is a retrovirus with a relatively stable genome, our findings indicate that it generates functionally relevant quasispecies in which specific variants with higher replication capacity may exhibit a selective advantage during transmission bottlenecks (27), potentially contributing to within-herd spread.

Our investigations provide functional and structural evidence for the regulatory roles of LTR175C and Tax69M/Tax73Q. However, the precise biochemical mechanisms underlying their context-dependent interaction have yet to be fully elucidated. Our western blot data confirmed that these single-residue substitutions do not alter total Tax protein expression levels or intracellular stability. Thus, the observed transactivation switching likely stems from qualitative conformational modulations. Specifically, it remains unknown whether these mutant Tax proteins exhibit altered binding affinities for host cellular partners, such as CREB (28), or whether the Tax/CREB complex differentially binds to the LTR175T versus LTR175C promoter topographies. Furthermore, these surface alterations may modulate the subsequent recruitment of coactivators or basal transcription factors, and further studies are necessary to clarify these fine-tuned mechanistic details. Nevertheless, our study does not fully recapitulate native LTR-driven Tax expression. It remains possible that the LTR175C versus LTR175T genotypes differentially influence Tax expression through altered CREB binding or transcription factor recruitment, or that the Tax mutations themselves affect expression when driven by the viral LTR. Future studies employing native LTR-driven Tax expression constructs in infected cells would be valuable to fully address these possibilities. Beyond these viral genetic factors, the phenotypic heterogeneity among strains with identical LTR and Tax genotypes points to the existence of additional genetic modifiers distributed across the viral genome, reinforcing the need for comprehensive whole-genome approaches to fully understand the genetic architecture of BLV viral fitness. Moreover, comprehensive transmission dynamics are inevitably influenced by multiple host and environmental variables. PVL is strongly affected by host immunogenetics, particularly BoLA-DRB3 polymorphisms (7, 29, 30). Because the current study was conducted on a single farm and did not fully model complex virus–host interactions, validation across broader epidemiological settings and integration with host immunological data (31–33) will be indispensable for confirming the general applicability of these findings.

In conclusion, although BLV transmission is strongly influenced by host factors (10), the virus may enhance its own fitness through coordinated interactions between the LTR and Tax mutations. These results underscore the importance of evaluating the genetic profile of circulating viruses, particularly mutations associated with HVP, alongside traditional host-based control strategies. Therefore, monitoring viral evolution is essential, as transmission-enhancing mutations may accumulate over time and require adaptive control measures.

## MATERIALS AND METHODS

### Samples

Blood samples were collected from all Holstein–Friesian cows on a single dairy farm in Tokyo, Japan. Sampling was conducted annually from 2013 to 2019 (herd size: 50–68), yielding 438 blood samples. To ensure statistical independence in the annual trend analyses, the sampling unit was standardized to one sample per animal per year. Thus, no individual cow contributed more than once to the data count within a given year. These 438 samples included 269 longitudinal samples obtained from the same individuals across different years. Additionally, 26 blood and tissue samples were collected from Japanese Black and Holstein–Friesian cows on different farms across Japan. Genomic DNA was extracted from whole blood and tissue samples using the Wizard Genomic DNA Purification Kit according to the manufacturer’s instructions (Promega, Madison, WI, USA).

### Virus genotyping

Substitutions at three loci (LTR175, Tax69, and Tax73) were genotyped using the rhAmp SNP Genotyping System (Integrated DNA Technologies, Coralville, IA, USA) following the manufacturer’s instructions. Briefly, the reaction mixture contained rhAmp Genotyping Master Mix, rhAmp Reporter Mix with ROX, and a custom rhAmp SNP Assay composed of allele- and locus-specific primers (Table S5). Real-time PCR was performed using a qTOWER³ G system (Analytik Jena, Jena, Germany), with results analyzed using qPCRsoft 4.1 (Analytik Jena).

### Farm-based genotype, haplotype, and PVL trend analyses

To evaluate the temporal dynamics of each polymorphic site, we analyzed the annual number of cattle carrying variant or wild-type alleles at LTR175, Tax69, and Tax73. As described above, the unit of analysis was strictly limited to one sample per animal per year, ensuring the statistical independence of observations within each cross-sectional annual population. For each locus, the Kendall rank correlation test was applied to assess trends in variant and wild-type frequencies across the study period (2013–2019). After excluding cows introduced from other farms, within-farm transmission trends were analyzed separately. To assess the temporal trends of major haplotypes, we calculated the annual frequencies of LTR175T/C and the four haplotypes (C-T-R, T-T-Q, T-T-R, and T-M-R) detected at the Tokyo farm during 2013–2019. Furthermore, the Kendall rank correlation test was used to evaluate trends in the relative proportion (percentage of total samples) for each haplotype. Additionally, linear regression analysis was used to estimate the annual rate of change (slope) for each relative proportion.

### Molecular clone and vector construction

Molecular clones were constructed into pSMART-LC Amp vector (Lucigen, Middleton, WI, USA) from blood samples of BLV-infected cattle according to a previously described method (13). Reporter virus-cloning molecular clones were generated by inserting a HiBiT-tag at the 3′-end of the *env* gene using molecular clones, as described previously (34). These constructions were determined using the ABI PRISM BigDye Terminator v3.1 Cycle Sequencing Kit (Applied Biosystems, Carlsbad, CA, USA) and an ABI PRISM 3130 Genetic Analyzer (Applied Biosystems) following a previously established protocol (13, 34). To measure transfection efficiency, we constructed a promoter- and enhancer-free firefly luciferase-cloning vector, pFL-null, which was generated from pRL-null (Toyo Ink, Tokyo, Japan) by replacing the *Renilla* luciferase (*RLuc*) gene with firefly luciferase (*FLuc*). The vector backbone and *FLuc* sequence were amplified via PCR using PrimeSTAR Max polymerase (TaKaRa Bio, Tokyo, Japan). The pRL-null vector lacking the *RLuc* sequence was amplified using the ΔRLuc primer pair. *FLuc* was amplified from pTK-Luc (Toyo Ink) using the FLuc primer pair. For constructing U3-cloned vectors (pU3-FLuc), the pTK-Luc vector lacking the thymidine promoter sequence was amplified using the ΔTK primer pair. U3 sequences from the AJ007 and AN006 strains were amplified using the U3 primer pair. For constructing HiBiT- and GS linker-tagged Tax expression vectors (pBApo-EF1α-Tax-HiBiT), the pBApo-EF1α Pur vector (TaKaRa Bio) was amplified using pBA primer pair (Table S6). Tax coding sequences from the AJ007 and AN006 strains were amplified using Tax primer pair (Table S6). Each PCR product was purified using Wizard SV Gel and the PCR Clean-Up System (Promega), followed by ligation using the In-Fusion HD Cloning Kit (TaKaRa Bio). Ligated vectors (wild-type/reporter molecular clones, pFL-null, pU3-FLuc, and pBApo-EF1α-Tax-HiBiT) were transformed into competent *Escherichia coli* strain Stbl3 or DH5α, respectively, for amplification. These vectors were subsequently purified using the PureYield Plasmid Midiprep System (Promega).

### Genetic analysis

BLV genomes were sequenced, as described previously (13). The whole-genome sequences of 54 strains identified in the present study were deposited in GenBank (accession numbers LC760082–LC760137; Table S1). To establish a robust consensus sequence, multiple sequence alignment of 115 sequences—comprising those identified in the present study and those reported previously (Table S1 and S2)—was performed using MAFFT v7.5 (35). A majority-rule consensus sequence was generated from these 115 sequences, with the most frequent nucleotide at each locus defined as the consensus residue.

### Measurement of viral productivity

RNA copy numbers of viruses produced from molecular clone-transfected 293T cells were determined using quantitative PCR, as previously reported (14). To determine transfection efficiency, the luciferase activity of the cells was measured using a Pikka Gene Kit (Toyo Ink), according to the manufacturer’s instructions. To eliminate bias due to transfection efficiency, viral RNA copy numbers were normalized against FLuc activity, according to a previous report (14).

### Induction of point mutations and recombination

Point mutations in the BLV genome and the U3 region in the LTR were introduced using previously described LTR175 mismatched primers (14) and Tax mismatched primers (Table S7). PCR amplification was performed using these mismatched primers with molecular clones or pU3-Luc as templates. The amplified PCR products were then ligated. Chimeric BLV molecular clones were constructed using the BLV molecular clones pBLVR-AJ008 and -AN006 as templates, as described previously (14). The pU3-Luc constructs carrying point mutations and molecular clones containing point mutations or recombination events were amplified in competent *E. coli* DH5α and Stbl3, respectively. These constructions were determined, as described above.

### Cells

Human embryonic kidney 293T cells and bovine mammary epithelial MAC-T cells were maintained in Dulbecco’s modified Eagle’s medium (Nissui Pharmaceutical Co., Ltd., Tokyo, Japan) supplemented with 10% fetal bovine serum. For MAC-T cells, the medium was further supplemented with 1 μg/ml hydrocortisone (Sigma-Aldrich, St. Louis, MO, USA).

### Measurement of NLuc activity in supernatants

Initially, 293T and MAC-T cells were transfected with reporter virus-cloning constructs together with pFL-null using FuGENE 4K (Promega). The transfected cells were washed twice, and fresh growth medium was added 24-h posttransfection. Supernatants were collected, and NLuc activity was measured using 20 µL of cell supernatant mixed with 20 µL of Nano-Glo HiBiT Lytic Detection System reagent (Promega). To evaluate transfection efficiency, FLuc activity was measured in transfected cells using the Pikka Gene Kit (Toyo Ink). Relative light units were quantified using a SpectraMax Mini luminometer (Molecular Devices, CA, USA).

### Prediction and analysis of protein structure

Protein structure prediction for Tax variants was performed using AlphaFold2 (v2.3.1; 36) installed on a local Ubuntu workstation. Predictions were generated using complete genetic databases with the monomer model, and the highest-ranked structures based on predicted local distance difference test scores were selected for further analysis. Structural superimposition and comparative analyses were performed using UCSF ChimeraX (v1.11.1; 37). The Matchmaker tool was employed to align the predicted structures of Tax variants (AJ007 and AN006) with the reference Tax (AF513), using the Needleman–Wunsch algorithm and BLOSUM-62 substitution matrix. RMSD values were calculated using Cα atoms to quantify structural similarity. These computed models represent predictions rather than experimentally determined structures and were used to formulate plausible structural hypotheses regarding potential alterations in electrostatic or hydrophobic interactions.

### Viral transactivation measurement

Each pU3-Luc construct together with pBApo-EF1α-CAGGS-Tax-HiBiT and pRL-null (Toyo Ink), was transfected into 293T cells using FuGENE 4K Transfection Reagent (Promega). The pU3-Luc and pRL-null plasmids were used to measure viral transactivation and transfection efficiency, respectively. At 48-h posttransfection, cells were analyzed for *FLuc* and RLuc activities using the Pikka Gene Dual Assay Kit (Toyo Ink).

### Western blot analysis

Western blot analysis was performed, as previously described (34). Briefly, collected 293T cells, which were transfected with pBApo-EF1α-Tax-HiBiT, were lysed in CelLytic M buffer (Sigma-Aldrich) and resolved in Laemmli sample buffer. The proteins (20 µg) were separated by 15% sodium dodecyl sulfate-polyacrylamide gel electrophoresis and transferred onto polyvinylidene fluoride membranes (Immobilon; Merck Millipore, Burlington, MA, USA). After blocking with 5% skim milk in TBST for 1 h, the membranes were incubated at 4°C overnight with anti-HiBiT tag (Promega) or anti-β-actin (Medical & Biological Laboratories Co., Ltd., Tokyo, Japan) antibodies. Subsequent secondary antibody incubation with horseradish peroxidase-conjugated anti-mouse/rabbit IgG (Jackson ImmunoResearch, West Grove, PA, USA) and chemiluminescent detection using Clarity Western ECL Substrate (Bio-Rad Laboratories, Hercules, CA, USA) were performed, as previously described (34). Signals were captured using an LAS 4000 mini image analyzer (Cytiva, Marlborough, MA, USA).

### PVL measurement

Quantitative PCR was performed using the qTOWER^3^ G system (Analytik Jena, Germany) to measure PVL, as described previously (38). PVL was determined using a standard curve based on the pBLVpur-FLK-GAPDH plasmid. For each sample, PVL was calculated by dividing the average number of BLV genomes by half the average number of GAPDH genomes, multiplying the result by 100,000. PVL was expressed as copies per 10^5^ cells. To compare PVL among the four haplotypes, values were analyzed using a linear mixed model. Pairwise comparisons between haplotypes were performed with Tukey adjustment for multiple testing.

### Statistical analyses

For all *in vitro* assays involving site-directed mutagenesis, including replication and transactivation analyses, statistical comparisons were strictly limited to planned paired evaluations between two distinct groups. Individual two-tailed Student’s *t*-tests were used to evaluate these specific experimental hypotheses. To ensure the mathematical validity of the parametric and nonparametric tests, the normality and homoscedasticity of all data sets were assessed using the Shapiro–Wilk test and the *F*-test, respectively. For the population-level comparison of viral productivity across a broad panel of field-derived original molecular clones, the distribution of viral RNA copy numbers deviated significantly from normality, confirming the appropriateness of the nonparametric Mann–Whitney *U* test (Wilcoxon rank-sum test). In contrast, for the mutagenesis assay data, both assumptions were fully satisfied, confirming the appropriateness of the Student’s *t*-test. For the *in vivo* PVL comparison among the four haplotypes (Fig. 4B), a linear mixed model was applied to account for years since entry and individual variation. Pairwise comparisons between haplotypes were then performed using Tukey’s honestly significant difference test to control the family-wise error rate across all six comparisons. All statistical analyses were performed in R v4.5.2 (39).

## Supplementary Material

Reviewer comments

## Data Availability

The whole-genome sequences of 54 BLV strains identified in the present study are available in GenBank under accession numbers LC760082–LC760137.

## SUPPLEMENTAL MATERIAL

Supplemental tables and figures spectrum.00869-26-s0001.pdf Tables S1 to S7 and Fig. S1 to S7.

OPEN PEER REVIEW reviewer-comments.pdf An accounting of the reviewer comments and feedback.

ASM does not own the copyrights to Supplemental Material that may be linked to, or accessed through, an article. The authors have granted ASM a non-exclusive, world-wide license to publish the Supplemental Material files. Please contact the corresponding author directly for reuse.
