# Supplementary material for: Combinations of long terminal repeat and tax protein substitutions influence bovine leukemia virus transmission through altered viral productivity
Source: Microbiol Spectr. 2026 Jul 27;14(9):e00869-26. doi: 10.1128/spectrum.00869-26 (PMC13532317; doi:10.1128/spectrum.00869-26)
Supplement: Supplementary file 1 [file reviewer-comments.pdf]

ASM | Microbiology Spectrum

Combinations of long terminal repeat and Tax protein substitutions influence bovine leukemia virus transmission through altered viral productivity

Hironobu Murakami, Reiichiro Sato, Jumpei Uchiyama, Kazuyuki Sogawa, Hiroho Ishida, and Makoto Nagai

Corresponding Author(s): Hironobu Murakami, Azabu Daigaku Jui Gakubu

Review Timeline:

Submission Date:	March 11, 2026
Editorial Decision:	April 22, 2026
Revision Received:	June 17, 2026
Editorial Decision:	June 29, 2026
Revision Received:	June 30, 2026
Accepted:	July 8, 2026

Editor: Hyun Jin Kwun

Reviewer(s): Disclosure of reviewer identity is with reference to reviewer comments included in decision letter(s). The following individuals involved in review of your submission have agreed to reveal their identity: Yoshinao Kubo (Reviewer #2)

Transaction Report:

(Note: With the exception of the correction of typographical or spelling errors that could be a source of ambiguity, letters and reports are not edited. The original formatting of letters and referee reports may not be reflected in this compilation.)

DOI: https://doi.org/10.1128/spectrum.00869-26

Re: Spectrum00869-26 (**Combinations of long terminal repeat and Tax protein substitutions influence bovine leukemia virus transmission through altered viral productivity**)

Dear Dr. Hironobu Murakami:

Thank you for the privilege of reviewing your work. Below you will find instructions from the Spectrum editorial office, and the reviewer comments.

Please return the manuscript within 60 days; if you cannot complete the modification within this time period, please contact me. If you do not wish to modify the manuscript and prefer to submit it to another journal, notify me immediately so that the manuscript may be formally withdrawn from consideration by Spectrum.

Revision Guidelines
To submit your modified manuscript, log into the submission site at https://spectrum.msubmit.net/cgi-bin/main.plex. Go to Author Tasks and click the appropriate manuscript title to begin. The information you entered when you first submitted the paper will be displayed; update this as necessary. Note the following requirements:

• Upload point-by-point responses to the issues raised by the reviewers in a file named "Response to Reviewers," NOT in your cover letter.
• Upload a compare copy of the manuscript (without figures) as a "Marked-Up Manuscript" file.
• Upload a clean .DOC/.DOCX version of the revised manuscript and remove the previous version.
• Each figure must be uploaded as a separate, editable, high-resolution file (TIFF or EPS preferred), and any multipanel figures must be assembled into one file.
• Any supplemental material intended for posting by ASM should be uploaded with their legends separate from the main manuscript. You can combine all supplemental material into one file (preferred) or split it into a maximum of 10 files with all associated legends included.

For complete guidelines on revision requirements, see our Submission and Review Process webpage. Submission of a paper that does not conform to guidelines may delay acceptance of your manuscript.

Data availability: ASM policy requires that data be available to the public upon online posting of the article, so please verify all links to sequence records, if present, and make sure that each number retrieves the full record of the data. If a new accession number is not linked or a link is broken, provide Spectrum production staff with the correct URL for the record. If the accession numbers for new data are not publicly accessible before the expected online posting of the article, publication may be delayed; please contact production staff (Spectrum@asmusa.org) immediately with the expected release date.

Publication Fees: For information on publication fees and which article types are subject to charges, visit our website. If your manuscript is accepted for publication and any fees apply, you will be contacted separately about payment during the production process; please follow the instructions in that e-mail. Arrangements for payment must be made before your article is published.

ASM Membership: Corresponding authors may join or renew ASM membership to obtain discounts on publication fees. Need to upgrade your membership level? Please contact Customer Service at Service@asmusa.org.

The ASM Journals program strives for constant improvement in our submission and publication process. Please tell us how we can improve your experience by taking this quick Author Survey.

Thank you for submitting your paper to Spectrum.

Sincerely,
Hyun Jin Kwun
Editor
Microbiology Spectrum

Reviewer #1 (Comments for the Author):

Overall, the manuscript presents a very interesting and well-structured study investigating how specific BLV mutations in the LTR and tax regions may influence viral productivity and may contribute to within-herd viral spread, although such dynamics are likely shaped by multiple viral, host, and environmental factors. The work is scientifically valuable, the experimental design is comprehensive, and the manuscript builds a coherent mechanistic story. The integration of longitudinal farm-based observations with molecular and functional analyses is a particular strength. However, there are several areas where additional clarification or

slightly more cautious wording may help further strengthen the manuscript and pre-empt potential reviewer concerns.

Major Comments

1. Interpretation of transmissibility may be somewhat overstated in several sections
Although the Discussion appropriately acknowledges that transmission dynamics are multifactorial and that broader validation is needed, several statements throughout the manuscript may still slightly overstate the extent to which the current data support direct inferences regarding transmissibility. Because within-herd spread is likely influenced not only by viral genotype, but also by host immunogenetic background, environmental conditions, and herd-management factors, it may be worth considering somewhat softer phrasing in places where "transmission advantage" or "high transmissibility" are stated more definitively. Alternative wording such as "associated with increased prevalence", "suggestive of enhanced transmission fitness", or "potentially contributing to within-herd spread" may provide more appropriate scientific caution.
Relevant examples include:
Introduction, lines 99-101: "...to identify substitutions with high transmissibility."
Results, lines 114-116: "These findings indicate that HVP confers a farm-level transmission advantage..."
Results, lines 217-222: "These results demonstrate that both HVP haplotypes have a clear advantage in within-farm horizontal transmission."
Discussion, lines 236-240 and 271-282

2. Clarification is needed regarding repeated longitudinal sampling in temporal analyses
It would be helpful to clarify how repeated longitudinal samples from the same animals were handled in the temporal trend analyses. As stated, 269 of the 438 total samples were collected longitudinally from the same individuals; however, it is currently unclear whether repeated samples from the same cows contributed multiple times to the annual genotype/haplotype counts. If so, this could introduce non-independence of observations and potentially affect the robustness of the trend analyses. A brief clarification of the unit of analysis (e.g., whether one sample per animal per year was used, or whether repeated measures were statistically accounted for) would strengthen the rigor and interpretability of this section.
Relevant sections:
Materials and Methods, lines 298-301
Materials and Methods, lines 398-408
Results, lines 104-116 and 200-222

3. Novelty relative to previous work may benefit from clearer emphasis
Because the authors have previously published related work on BLV mutations affecting viral productivity (e.g., DOI: 10.1016/j.virol.2019.08.015), it may be helpful to more explicitly highlight the novelty of the current study relative to prior publications. At present, the manuscript references previous findings but could more clearly distinguish what specifically is newly demonstrated here (e.g., long-term in vivo herd dynamics, combined haplotype analyses, identification of compensatory Tax mutations, etc.). Strengthening this distinction may help pre-empt reviewer concerns regarding overlap with earlier studies.

Minor Comments

1. Introduction wording may benefit from slightly more cautious phrasing regarding transmissibility
Overall, the Introduction is strong and well structured. One general suggestion is that some statements regarding "transmissibility" may read slightly stronger than warranted before the transmission-related analyses are presented. In several places, softer phrasing such as "transmission potential," "transmission-associated traits," or "enhanced transmission fitness" may improve scientific caution and reduce the risk of reviewer concerns regarding overinterpretation.

2. Structural/mechanistic interpretation based on AlphaFold may be somewhat overstated
The structural analysis is highly interesting; however, some mechanistic interpretations may read slightly stronger than warranted for AlphaFold-based modeling alone. Statements suggesting that the mutations alter viral productivity "through structural changes" or directly disrupt electrostatic/hydrophobic interactions may be interpreted as mechanistic conclusions rather than structural hypotheses. It may therefore be helpful to slightly soften the wording and frame these as predicted or plausible structural effects unless additional experimental structural validation is available.
Relevant sections:
Materials and Methods, lines 367-376
Results, lines 152-167
Discussion, lines 241-243

3. Statistical methods for in vitro analyses may require greater detail
The statistical methods section may benefit from slightly more detail regarding the in vitro analyses. At present, only Student's t-test is mentioned; however, the Results include multiple comparisons across strains, mutants, and experimental conditions. It may therefore be useful to specify more clearly which analyses used t-tests, whether multiple-testing correction was applied where appropriate, and whether assumptions for parametric testing were assessed.
Relevant sections:
Materials and Methods, lines 420-422

Results, lines 121-129 and 170-190

4. Several figures would benefit from improved readability before submission. In particular, the font size of axis labels, strain names, and significance markings appears quite small, especially in Figures 1 and 4, and may be difficult to read at journal print scale. Enlarging these elements and slightly reducing visual crowding would improve clarity.
5. Figure 2 contains several different types of structural information in a single layout, which makes the figure somewhat difficult to interpret quickly. It may help to simplify the arrangement of panels and more clearly mark the exact residues or local structural features that support the main conclusions

Overall, this is a compelling and thoughtfully designed manuscript that presents an interesting mechanistic and epidemiological investigation into BLV genetic variation. With several relatively minor clarifications and some moderation of wording/interpretation, the manuscript could be further strengthened and made more robust against likely reviewer critiques.

Reviewer #2 (Comments for the Author):

Murakami et al. show that a cytosine residue at nucleotide position 175 of the LTR (LTR175C) enhances viral production compared with a thymidine at the same position (LTR175T). However, this effect is abrogated by amino acid substitutions at positions 69 and 73 in the Tax protein. Although this finding is interesting, it remains preliminary and has several technical issues as described below:

1. BLV naturally proliferates in cattle, not in humans. Because the virus relies on many host factors for replication, bovine cells should be used in this study. The use of human 293T cells may alter the outcome.

2. In Figure 1, viral production was compared between original clones containing 175T and their LTR T175C mutants or between original clones containing 175C and their LTR C175T mutants. Viral production involves many viral steps including transcription, translation, processing, and assembly, and each clone may harbor additional mutations that affect these steps. Therefore, viral production should be directly compared between 175T-containing and 175C-containing original clones.

3. Recombinant viruses between AJ007 and AN006 were constructed in this study. However, both clones lack responsiveness to LTR 175C. Generally, recombinants should be constructed between a non-responsive clone and a response clone to determine the region responsible for the responsiveness to LTR 175C. If AJ007 and AN006 encode Tax proteins with identical sequences, the responsible region cannot be defined using these recombinants.

4. Tax activates transcription from LTR. However, analyses of viral production and transcriptional activity were performed only in the presence of Tax. Because mutants at positions 69 and 73 may affect Tax-mediated transcriptional activation, transcriptional activity should be measured both in the absence and presence of Tax to clarify their effects on transcriptional activation.

5. This study remains incomplete due to the lack of mechanistic insight into how the AJ007 and AN006 Tax proteins do not respond to LTR 175C. Tax activates LTR transcription through interaction with CREB. It is therefore important to determine whether these Tax variants have lost their ability to interact with CREB, whether Tax protein levels are reduced, or whether the transcriptional complex including Tax and CREB can bind to both LTR 175T and 175C. Mechanistic evidence addressing these possibilities is required.

Overall, the manuscript presents a very interesting and well-structured study investigating how specific BLV mutations in the LTR and tax regions may influence viral productivity and may contribute to within-herd viral spread, although such dynamics are likely shaped by multiple viral, host, and environmental factors. The work is scientifically valuable, the experimental design is comprehensive, and the manuscript builds a coherent mechanistic story. The integration of longitudinal farm-based observations with molecular and functional analyses is a particular strength. However, there are several areas where additional clarification or slightly more cautious wording may help further strengthen the manuscript and pre-empt potential reviewer concerns.

Major Comments

1. Interpretation of transmissibility may be somewhat overstated in several sections
Although the Discussion appropriately acknowledges that transmission dynamics are multifactorial and that broader validation is needed, several statements throughout the manuscript may still slightly overstate the extent to which the current data support direct inferences regarding transmissibility. Because within-herd spread is likely influenced not only by viral genotype, but also by host immunogenetic background, environmental conditions, and herd-management factors, it may be worth considering somewhat softer phrasing in places where "transmission advantage" or "high transmissibility" are stated more definitively. Alternative wording such as *"associated with increased prevalence"*, *"suggestive of enhanced transmission fitness"*, or *"potentially contributing to within-herd spread"* may provide more appropriate scientific caution.
Relevant examples include:

- Introduction, lines 99–101: "…to identify substitutions with high transmissibility."
- Results, lines 114–116: "These findings indicate that HVP confers a farm-level transmission advantage…"
- Results, lines 217–222: "These results demonstrate that both HVP haplotypes have a clear advantage in within-farm horizontal transmission."
- Discussion, lines 236–240 and 271–282

2. Clarification is needed regarding repeated longitudinal sampling in temporal analyses
It would be helpful to clarify how repeated longitudinal samples from the same animals were handled in the temporal trend analyses. As stated, 269 of the 438 total samples were collected longitudinally from the same individuals; however, it is currently unclear whether repeated samples from the same cows contributed multiple times to the annual genotype/haplotype counts. If so, this could introduce non-independence of observations and potentially affect the robustness of the trend analyses. A brief clarification of the unit of analysis (e.g., whether one sample per animal per year was used, or whether repeated measures were statistically accounted for) would strengthen the rigor and interpretability of this section.
Relevant sections:

- Materials and Methods, lines 298–301
- Materials and Methods, lines 398–408
- Results, lines 104–116 and 200–222

3. Novelty relative to previous work may benefit from clearer emphasis
Because the authors have previously published related work on BLV mutations affecting viral productivity (e.g., DOI: 10.1016/j.virol.2019.08.015), it may be helpful to more explicitly highlight the novelty of the current study relative to prior publications. At present, the manuscript references previous findings but could more clearly distinguish what specifically is newly demonstrated here (e.g., long-term in vivo herd dynamics, combined haplotype analyses, identification of compensatory Tax mutations, etc.). Strengthening this distinction may help pre-empt reviewer concerns regarding overlap with earlier studies.

Minor Comments

1. Introduction wording may benefit from slightly more cautious phrasing regarding transmissibility
Overall, the Introduction is strong and well structured. One general suggestion is that some statements regarding "transmissibility" may read slightly stronger than warranted before the transmission-related analyses are presented. In several places, softer phrasing such as *"transmission potential," "transmission-associated traits,"* or *"enhanced transmission fitness"* may improve scientific caution and reduce the risk of reviewer concerns regarding overinterpretation.

2. Structural/mechanistic interpretation based on AlphaFold may be somewhat overstated
The structural analysis is highly interesting; however, some mechanistic interpretations may read slightly stronger than warranted for AlphaFold-based modeling alone. Statements suggesting that the mutations alter viral productivity *"through structural changes"* or directly disrupt electrostatic/hydrophobic interactions may be interpreted as mechanistic conclusions rather than structural hypotheses. It may therefore be helpful to slightly soften the wording and frame these as predicted or plausible structural effects unless additional experimental structural validation is available.
Relevant sections:

- Materials and Methods, lines 367–376
- Results, lines 152–167
- Discussion, lines 241–243

3. Statistical methods for in vitro analyses may require greater detail
The statistical methods section may benefit from slightly more detail regarding the in vitro analyses. At present, only Student's *t*-test is mentioned; however, the Results include multiple comparisons across strains, mutants, and experimental conditions. It may therefore be useful to specify more clearly which analyses used *t*-tests, whether multiple-testing correction was applied where appropriate, and whether assumptions for parametric testing were assessed.
Relevant sections:

- Materials and Methods, lines 420–422
- Results, lines 121–129 and 170–190

4. Several figures would benefit from improved readability before submission. In particular, the font size of axis labels, strain names, and significance markings appears quite small, especially in Figures 1 and 4, and may be difficult to read at journal print scale. Enlarging these elements and slightly reducing visual crowding would improve clarity.

5. Figure 2 contains several different types of structural information in a single layout, which makes the figure somewhat difficult to interpret quickly. It may help to simplify the arrangement of panels and more clearly mark the exact residues or local structural features that support the main conclusions

Overall, this is a compelling and thoughtfully designed manuscript that presents an interesting mechanistic and epidemiological investigation into BLV genetic variation. With several relatively minor clarifications and some moderation of wording/interpretation, the manuscript could be further strengthened and made more robust against likely reviewer critiques.

We sincerely thank both reviewers for their highly encouraging, insightful, and constructive assessments of our manuscript. We are grateful to Reviewer #1 for recognizing the value of our study as a compelling and thoughtfully designed mechanistic and epidemiological investigation of BLV genetic variation and for providing excellent guidance on statistical methodology, figure readability, and scientific caution in phrasing, particularly regarding AlphaFold-based structural modeling and transmissibility-related terminology. We are equally grateful to Reviewer #2 for their rigorous and critical assessment of our experimental design. This prompted us to validate our key findings in bovine cells, expand our direct comparative analysis to 109 field-derived clones, and provide additional mechanistic data, including western blot analysis and Tax-independent controls. The valuable input from both reviewers has led to a substantial revision of the manuscript. The revised sentences are shown in red in the revised manuscript. We believe that these changes have significantly strengthened the robustness, clarity, and biological relevance of our work.

Response to Reviewer #1

Major Comments

1. Interpretation of transmissibility may be somewhat overstated in several sections

Although the Discussion appropriately acknowledges that transmission dynamics are multifactorial and that broader validation is needed, several statements throughout the manuscript may still slightly overstate the extent to which the current data support direct inferences regarding transmissibility. Because within-herd spread is likely influenced not only by viral genotype, but also by host immunogenetic background, environmental conditions, and herd-management factors, it may be worth considering somewhat softer phrasing in places where "transmission advantage" or "high transmissibility" are stated more definitively. Alternative wording such as "associated with increased prevalence", "suggestive of enhanced transmission fitness", or "potentially contributing to within-herd spread" may provide more appropriate scientific caution.

Relevant examples include:

Introduction, lines 99-101: "...to identify substitutions with high transmissibility."

Results, lines 114-116: "These findings indicate that HVP confers a farm-level transmission advantage..."

Results, lines 217-222: "These results demonstrate that both HVP haplotypes have a clear advantage in within-farm horizontal transmission."

Discussion, lines 236-240 and 271-282

Answer: We sincerely appreciate your constructive and insightful comment. We agree that within-herd transmission dynamics are multifactorial and shaped by the interplay among viral genotypes, host immunogenetic backgrounds, and various environmental or management factors. As suggested, we have carefully revised and softened our phrasing throughout the manuscript to exercise appropriate scientific caution (Introduction, lines 106–108; Results, lines 121–123 and 276–281; Discussion, lines 296–297 and 343–347). All definitive expressions regarding "transmissibility" or "transmission advantage" have been modified to incorporate your helpful recommendations (e.g., using terms such as "transmission potential," "enhanced transmission fitness," and "associated with").

2. Clarification is needed regarding repeated longitudinal sampling in temporal analyses
It would be helpful to clarify how repeated longitudinal samples from the same animals were handled in the temporal trend analyses. As stated, 269 of the 438 total samples were collected longitudinally from the same individuals; however, it is currently unclear whether repeated samples from the same cows contributed multiple times to the annual genotype/haplotype counts. If so, this could introduce non-independence of observations and potentially affect the robustness of the trend analyses. A brief clarification of the unit of analysis (e.g., whether one sample per animal per year was used, or whether repeated measures were statistically accounted for) would strengthen the rigor and interpretability of this section.
Relevant sections:
Materials and Methods, lines 298-301
Materials and Methods, lines 398-408
Results, lines 104-116 and 200-222

Answer: We agree that ensuring the independence of observations is crucial for the validity of the temporal trend analyses. We appreciate this opportunity to clarify our data-handling procedure, which preserves the statistical robustness of the reported findings. In our study, the unit of analysis for the annual genotype and haplotype counts was strictly defined as one sample per animal per year. Although 269 samples were obtained longitudinally from the same individuals across different years, no individual animal was counted more than once within a given year. Therefore, in any given year, the annual counts represent a cross-sectional snapshot of the herd population, with observations

being completely independent. Regarding the multiyear longitudinal tracking, these repeated measures over time reflect the actual long-term persistence of and new infections with viral strains within the closed farm population. In the time series trend analyses (Kendall's τ and linear regression), each year's overall haplotype proportion was treated as a single population-level metric. Because intra-year inflation of specific animal data was strictly avoided, the longitudinal nature of herd management does not artificially skew annual counts or compromise the robustness of the statistical trends. To make this explicit to readers, we added a sentence clarifying the unit of analysis in the revised Results (lines 112–123 and 268–270) and Materials and Methods sections (lines 378–382 and 399–402). Thank you for this valuable comment. It has helped us improve the clarity and rigor of our methodology.

3. Novelty relative to previous work may benefit from clearer emphasis
Because the authors have previously published related work on BLV mutations affecting viral productivity (e.g., DOI: 10.1016/j.virol.2019.08.015), it may be helpful to more explicitly highlight the novelty of the current study relative to prior publications. At present, the manuscript references previous findings but could more clearly distinguish what specifically is newly demonstrated here (e.g., long-term in vivo herd dynamics, combined haplotype analyses, identification of compensatory Tax mutations, etc.). Strengthening this distinction may help pre-empt reviewer concerns regarding overlap with earlier studies.

Answer: We are grateful for your highly constructive recommendation. We agree that explicitly distinguishing the novelty of the present study from our previous work (*Virology*, 2019) will strengthen the manuscript.

Our 2019 paper focused primarily on the *in vitro* characterization of the single LTR175C mutation. In contrast, the current study fundamentally advances this knowledge by shifting the paradigm from a single locus to combined multilocus haplotypes and validating their epidemiological relevance in the field. As suggested, we have significantly revised and strengthened the emphasis on our study's novelty in the Introduction (lines 101–108) and Discussion sections (lines 310–316).

Minor Comments
1. Introduction wording may benefit from slightly more cautious phrasing regarding

transmissibility
Overall, the Introduction is strong and well structured. One general suggestion is that some statements regarding "transmissibility" may read slightly stronger than warranted before the transmission-related analyses are presented. In several places, softer phrasing such as "transmission potential," "transmission-associated traits," or "enhanced transmission fitness" may improve scientific caution and reduce the risk of reviewer concerns regarding overinterpretation.

Answer: We sincerely thank you for this encouraging and thoughtful suggestion. We agree that introducing these concepts with appropriate scientific caution is vital before presenting our data and transmission-related analyses. Following your recommendations, we have carefully reviewed the Introduction section and modified the terminology throughout to adopt softer, more precise phrasing. Specifically, we have replaced definitive references to "transmissibility" with the alternative terms you suggested, such as "transmission potential," "transmission-associated traits," and "transmission fitness."

2. Structural/mechanistic interpretation based on AlphaFold may be somewhat overstated
The structural analysis is highly interesting; however, some mechanistic interpretations may read slightly stronger than warranted for AlphaFold-based modeling alone. Statements suggesting that the mutations alter viral productivity "through structural changes" or directly disrupt electrostatic/hydrophobic interactions may be interpreted as mechanistic conclusions rather than structural hypotheses. It may therefore be helpful to slightly soften the wording and frame these as predicted or plausible structural effects unless additional experimental structural validation is available.
Relevant sections:
Materials and Methods, lines 367-376
Results, lines 152-167
Discussion, lines 241-243

Answer: We completely agree with your excellent point. Although AlphaFold2 provides valuable structural models to guide our understanding, these are computational predictions, not experimentally determined structures (such as those from X-ray crystallography or cryo-EM). Therefore, framing these insights as definitive physical mechanisms rather than structural hypotheses warrants appropriate scientific caution. Per your suggestion, we have thoroughly revised the text in the Materials and Methods (lines

494–497), Results (lines 188–213), and Discussion sections (lines 296–297) to soften our phrasing. We have systematically reframed our interpretations using more cautious terminology, such as “predicted,” “suggested,” “potentially mediated by,” and “modeled to,” to present these structural effects as plausible hypotheses.

3. Statistical methods for in vitro analyses may require greater detail

The statistical methods section may benefit from slightly more detail regarding the in vitro analyses. At present, only Student's *t*-test is mentioned; however, the Results include multiple comparisons across strains, mutants, and experimental conditions. It may therefore be useful to specify more clearly which analyses used t-tests, whether multiple-testing correction was applied where appropriate, and whether assumptions for parametric testing were assessed.

Relevant sections:

Materials and Methods, lines 420-422

Results, lines 121-129 and 170-190

Answer: We appreciate the rigorous attention paid to our statistical methodology. For all *in vitro* analyses (Figures 1B, 1C, 1D, 2A, 3A, 3B, 3C, and 3D), experiments were designed strictly as independent, planned comparisons between two distinct groups (1 vs. 1 pair), for which Student’s *t* t-test or Mann–Whitney *U* test is mathematically correct and appropriate. Although multiple strains or constructs are displayed within a single figure panel for concise visualization, they were not compared with one another simultaneously. To prevent any misunderstanding among readers, we have clearly described in the Results section that experiments involved two-group comparisons (lines 140–142 and 159–174). We have also verified that the variances and distributions within these independent pairs met the standard assumptions for parametric testing, and we have expanded the Statistical analyses subsection in the Materials and Methods to clearly outline the nature of our testing. For the *in vivo* PVL comparisons among haplotypes (Fig. 4B and Table S4), we analyzed four haplotype groups simultaneously. In this case, we appropriately applied a linear mixed model followed by Tukey’s honestly significant difference test for all six pairwise comparisons to control the family-wise error rate. This approach is clearly described in the Materials and Methods (lines 533–548) and the full results are presented in Table S4.

4. Several figures would benefit from improved readability before submission. In particular, the font size of axis labels, strain names, and significance markings appears quite small, especially in Figures 1 and 4, and may be difficult to read at journal print scale. Enlarging these elements and slightly reducing visual crowding would improve clarity.

Answer: Per your recommendation, we have thoroughly revised our figures, paying particular attention to Figures 1 and 4, to enhance their clarity and reduce visual crowding. Specifically, we implemented the following modifications:

1. Font-size enlargement: We substantially increased the font sizes across all panels.
2. Significance markings: The text for significance markings (e.g., *, **, and NS) was enlarged and positioned with a larger clearance above the error bars to prevent blending into the graph lines.
3. Reduction of visual crowding: For Figure 1, we adjusted the bar spacing and width so that the individual strain labels would be easier to read. For Figure 4, we optimized the layout of the longitudinal trends and PVL plots to ensure maximum readability.

We have applied these readability standards consistently to all figures in the revised manuscript. We believe that these visual adjustments have substantially improved the presentation and professional quality of our study.

5. Figure 2 contains several different types of structural information in a single layout, which makes the figure somewhat difficult to interpret quickly. It may help to simplify the arrangement of panels and more clearly mark the exact residues or local structural features that support the main conclusions.

Answer: We agree that combining the phenotypic chimera assay with three distinct types of computational structural renderings (ribbon overlays, electrostatic potential maps, and hydrophobicity surfaces) within a single figure created excessive visual density, hindering immediate interpretation.

To comprehensively address your concern and improve the scannability of our data, we have reorganized our presentation by splitting the original figure into a focused main Figure 2 and a detailed Supplementary Figure S4:

1. Simplification of main Figure 2: The revised Figure 2 now functions as a clear,

streamlined visual summary of our core findings. It pairs our wet-lab chimeric assay (panel A) directly with the localized surface property models (panels B, C, and D) that illustrate the shifts in electrostatic and hydrophobic surface properties. Unnecessary layers have been removed to enable readers to quickly grasp the genotype-phenotype correlations.

2. Transfer of detailed backbones to Figure S4: The complex, fine-grained structural alignments and ribbon overlays generated by AlphaFold2 (including zoomed-in views of positions Tax69 and Tax73) have been moved to the newly designated Figure S4. This ensures that the technical, high-resolution validation data remain accessible to readers who are structural specialists without cluttering the main manuscript figure.
3. Enhanced visual cues and marking: Across both revised Figure 2 and the new Figure S4, we have enlarged the text labels, emphasized target residue indicators (Tax69, Tax73, and V146) using high-contrast arrows, and highlighted local electrostatic/hydrophobic pockets with distinct color-coded outlines to instantly guide the reader's attention to the features supporting our mechanistic hypotheses.

Reviewer #2 (Comments for the Author):

Murakami et al. show that a cytosine residue at nucleotide position 175 of the LTR (LTR175C) enhances viral production compared with a thymidine at the same position (LTR175T). However, this effect is abrogated by amino acid substitutions at positions 69 and 73 in the Tax protein. Although this finding is interesting, it remains preliminary and has several technical issues as described below:

1. BLV naturally proliferates in cattle, not in humans. Because the virus relies on many host factors for replication, bovine cells should be used in this study. The use of human 293T cells may alter the outcome.

Answer: We agree with your critically important point. BLV is a bovine retrovirus. Thus, its replication and regulatory dynamics depend heavily on host-specific factors. Therefore, experimental outcomes in human-derived cells (such as 293T cells) require careful validation in a biologically relevant bovine host system to confirm their generalizability. To directly address your concern and verify the biological validity of our findings, we selected infectious molecular clones representing both highly responsive strains (AJ017 and AJ013) and the specific variant strains (AJ007 and AN006) based on our initial 293T screening. We then transfected these parental wild-type clones and their corresponding LTR 175 mutants into MAC-T cells (an established bovine mammary epithelial cell line). As indicated in the newly added Figure S1 (and described in the revised Results and Materials and Methods sections), the experimental outcomes in bovine MAC-T cells recapitulated our initial observations in 293T cells as follows:

1. In the responsive backgrounds, introducing the T175C mutation into the LTR175T strain (AJ017) significantly enhanced viral production (Figure S1A), while the reverse C175T mutation in the LTR175C strain (AJ013) markedly suppressed viral production (Figure S1B).
2. Crucially, the nonresponsive strains (AJ007 and AN006) remained unaffected by the LTR175 substitutions, even within the bovine intracellular environment, exhibiting no significant differences in viral production ($p > 0.05$).

These cross-species validation data demonstrate that the functional interaction between the LTR175 locus and the Tax genetic background is an intrinsic viral property independent of human-specific host factors. We believe that this robust confirmation in bovine cells significantly strengthens our manuscript’s biological relevance and reliability.

2. In Figure 1, viral production was compared between original clones containing 175T and their LTR T175C mutants or between original clones containing 175C and their LTR C175T mutants. Viral production involves many viral steps including transcription, translation, processing, and assembly, and each clone may harbor additional mutations that affect these steps. Therefore, viral production should be directly compared between 175T-containing and 175C-containing original clones.

Answer: We agree with your insightful assessment. Retroviral replication encompasses multiple orchestrated steps (including transcription, translation, processing, and particle assembly). Thus, individual viral clones isolated from the field inevitably possess distinct sets of synonymous or nonsynonymous passenger mutations across their genomes. Such variations may potentially act as confounders in single-strain reverse genetics comparisons. To rigorously address this concern and definitively isolate the intrinsic functional impact of the LTR175 locus from unique genomic background noise, we substantially expanded our analysis by performing a large-scale direct comparison using a comprehensive panel comprising 109 distinct field-derived BLV original infectious molecular clones (56 newly cloned strains from our active surveillance across Japan and 53 previously reported isolates, excluding deletion variants). We categorized these 109 natural variants into two groups based on their native LTR genotype (those naturally harboring LTR175T vs. those naturally harboring LVP-associated LTR175C) and directly quantified their baseline viral RNA copy numbers using quantitative PCR.

As demonstrated in revised Figure 1B (and integrated into the revised Results section), this macro-level direct comparison yielded the following exceptionally clear and robust outcome:

- Despite the natural genetic diversity and potential unique passenger mutations scattered across the 109 independent viral backgrounds, the original strains naturally harboring LTR175C exhibited a significantly higher level of baseline viral production as a population compared with those harboring LTR175T (***$p < 0.001$).

By scaling up our direct comparison to over 100 field-derived original clones, we statistically neutralized individual genetic background variations. This macro-level validation confirmed that the enhancing effect of the LTR175C residue on viral

productivity is a robust, population-wide property of BLV, transcending the specific genetic background of any single isolated clone and directly supporting its evolutionary dominance on the farm. We believe this extensive direct verification fully resolves your concern and considerably elevates the epidemiological and mechanistic robustness of our study.

3. Recombinant viruses between AJ007 and AN006 were constructed in this study. However, both clones lack responsiveness to LTR 175C. Generally, recombinants should be constructed between a non-responsive clone and a response clone to determine the region responsible for the responsiveness to LTR 175C. If AJ007 and AN006 encode Tax proteins with identical sequences, the responsible region cannot be defined using these recombinants.

Answer: We deeply appreciate your critical and logically sound comment. We agree that under the general convention of reverse genetics, mapping a specific responsive domain is typically achieved by crossing a responsive clone with a nonresponsive clone. First, to resolve a fundamental premise regarding the viral sequences, we respectfully clarify that AJ007 and AN006 do not encode identical Tax proteins. As described in our manuscript, their Tax proteins differ at the two novel amino acid substitutions identified in this study (located at amino acid residues 69 and 73 of the Tax protein). In contrast, the precise genetic determinants causing the baseline differences in viral production between these two strains remain unidentified. Based on our experiments conducted to date, we hypothesized that these unrecognized substitutions affect viral production independently of the LTR175 substitution.

Our strategic rationale for this whole-virus chimeric experiment (Figure 2A) was rooted in a highly intriguing phenotypic paradox observed between these two distinct field isolates regarding their baseline viral productivity:

1. AJ007 naturally harbors LTR175T (typically a low-productivity genotype), yet unexpectedly exhibits high baseline levels of viral production.
2. AN006 naturally harbors LTR175C (typically a high-productivity genotype), yet paradoxically exhibits low baseline levels of viral production.

This profound phenotypic inversion led us to strongly hypothesize that there must be critical, LTR175-independent regulatory mutations must exist within the broader viral

genome background (beyond the LTR175 locus) that can override or modulate the expected LTR-driven viral production. Thus, the large-scale chimeric viruses in Figure 2A were specifically constructed to investigate, at a genome-wide level, which genomic regions harbor non-LTR background variations that drive or suppress total virus production. Crucially, this genome-wide characterization serves as an essential foundation for investigating the subsequent relationship between the new substitutions of Tax protein and substitution at LTR175 (Figure 3). Instead of relying solely on the whole-virus chimeras to define the mechanism, we followed up this initial genomic screening with site-directed mutagenesis and transactivation (luciferase reporter) assays (Figures 3C and 3D).

We recognize that our original manuscript did not sufficiently explain this experimental rationale and chronological flow. To address this and prevent further ambiguity, we have thoroughly revised the corresponding Results section to clearly articulate our strategic intentions, the nonidentical nature of the Tax proteins, and the unique phenotypic backgrounds of AJ007 and AN006.

4. Tax activates transcription from LTR. However, analyses of viral production and transcriptional activity were performed only in the presence of Tax. Because mutants at positions 69 and 73 may affect Tax-mediated transcriptional activation, transcriptional activity should be measured both in the absence and presence of Tax to clarify their effects on transcriptional activation.

Answer: We agree with your valuable suggestion that measuring the transcriptional activity in both the absence and presence of Tax is essential to distinguish whether the observed mutational effects are specifically associated with Tax-mediated transactivation or merely reflect alterations in basal, Tax-independent transcription. In accordance with your recommendation, we have comprehensively addressed this point by including the transactivation data in the complete absence of Tax (indicated as "–" in Figures 3C and 3D) using an empty vector control.

As shown in the updated Figures 3C and 3D:

1. In the absence of Tax ("–"), the basal transcriptional activities of the LTR175T and LTR175C promoters were extremely low and remained equivalent, showing no statistically significant differences regardless of the LTR genotypes or the viral

backgrounds.

2. In the presence of Tax, the WT Tax proteins of AJ007 and AN006 did not show a preference for the LTR175C promoter. However, the introduction of the specific single-residue mutations (Q73R in AJ007 and M69T in AN006) specifically triggered a significant, selective increase in transactivation activity, directed toward the LTR175C promoter.

These contrasting observations provide strong evidence that the altered promoter responsiveness observed with the Tax mutants is not the result of baseline, Tax-independent transcriptional changes. Instead, it is strictly governed by the functional interplay during Tax-mediated transactivation in a site-specific manner.

We believe that the addition of these “absence of Tax” control data has substantially strengthened the mathematical and biological validity of our conclusions. The revised Figures 3C and 3D, along with the corresponding descriptions in the Results and Discussion sections, have been updated accordingly.

5. This study remains incomplete due to the lack of mechanistic insight into how the AJ007 and AN006 Tax proteins do not respond to LTR 175C. Tax activates LTR transcription through interaction with CREB. It is therefore important to determine whether these Tax variants have lost their ability to interact with CREB, whether Tax protein levels are reduced, or whether the transcriptional complex including Tax and CREB can bind to both LTR 175T and 175C. Mechanistic evidence addressing these possibilities is required.

Answer: We agree that uncovering the precise biochemical mechanisms, such as the physical interaction with CREB or the binding affinity of the Tax/CREB complex to the LTR175T/175C promoters, is crucial to fully elucidate BLV transactivation dynamics. To fundamentally address this concern, we performed western blot analysis to determine whether the observed functional differences could be explained by altered Tax protein expression or stability. As evident in the updated Figure 3E, the introduction of the single-residue mutations (Q73R in AJ007 and M69T in AN006) did not alter Tax protein expression levels or intracellular stability, with both variants maintaining robust, equivalent bands comparable to their respective wild-types. This finding rules out the possibility that the transactivation changes (Figures 3C and 3D) were simply due to reduced protein expression or accelerated degradation of the mutant Tax proteins.

Furthermore, it demonstrates the following:

1. The mutations at residues 69 and 73 impact the qualitative transactivation efficiency of the Tax protein rather than its quantitative expression.
2. The site-specific alterations in LTR promoter preference are probably governed by functional conformational changes or localized interactive dynamics during complex formation on the LTR.

We agree that the essential next step is determining whether these Tax variants have lost their ability to interact with CREB or whether the Tax/CREB complex exhibits differential binding affinity for LTR175T versus LTR175C. Accordingly, we explicitly expanded the Discussion section (lines 302–303 and 348–365) to acknowledge these unanswered questions, framing CREB interaction assays and electrophoretic mobility shift assays as the logical and immediate extensions of the present study, which we are actively pursuing. We believe that the addition of the data in Figure 3E provides a solid mechanistic baseline that substantially elevates the scientific rigor of our manuscript, while the revised Discussion clearly outlines the path forward, thereby fully addressing your mechanistic concerns.

Re: Spectrum00869-26R1 (**Combinations of long terminal repeat and Tax protein substitutions influence bovine leukemia virus transmission through altered viral productivity**)

Dear Dr. Hironobu Murakami:

Thank you for the privilege of reviewing your work. Your manuscript has been editorially accepted for publication. However, there are a few additional questions in the submission form that need to be answered before the final decision. Once these are completed, please return your submission so that I can move your paper forward to acceptance. Below you will find instructions from the Spectrum editorial office, and the reviewer comments.

Please return the manuscript within 60 days; if you cannot complete the modification within this time period, please contact me. If you do not wish to modify the manuscript and prefer to submit it to another journal, notify me immediately so that the manuscript may be formally withdrawn from consideration by Spectrum.

Revision Guidelines
To submit your modified manuscript, log into the submission site at https://spectrum.msubmit.net/cgi-bin/main.plex. Go to Author Tasks and click the appropriate manuscript title to begin. The information you entered when you first submitted the paper will be displayed; update this as necessary. Note the following requirements:

• Upload point-by-point responses to the issues raised by the reviewers in a file named "Response to Reviewers," NOT in your cover letter.
• Upload a compare copy of the manuscript (without figures) as a "Marked-Up Manuscript" file.
• Upload a clean .DOC/.DOCX version of the revised manuscript and remove the previous version.
• Each figure must be uploaded as a separate, editable, high-resolution file (TIFF or EPS preferred), and any multipanel figures must be assembled into one file.
• Any supplemental material intended for posting by ASM should be uploaded with their legends separate from the main manuscript. You can combine all supplemental material into one file (preferred) or split it into a maximum of 10 files with all associated legends included.

For complete guidelines on revision requirements, see our Submission and Review Process webpage. Submission of a paper that does not conform to guidelines may delay acceptance of your manuscript.

Data availability: ASM policy requires that data be available to the public upon online posting of the article, so please verify all links to sequence records, if present, and make sure that each number retrieves the full record of the data. If a new accession number is not linked or a link is broken, provide Spectrum production staff with the correct URL for the record. If the accession numbers for new data are not publicly accessible before the expected online posting of the article, publication may be delayed; please contact production staff (Spectrum@asmusa.org) immediately with the expected release date.

Publication Fees: For information on publication fees and which article types are subject to charges, visit our website. If your manuscript is accepted for publication and any fees apply, you will be contacted separately about payment during the production process; please follow the instructions in that e-mail. Arrangements for payment must be made before your article is published.

ASM Membership: Corresponding authors may join or renew ASM membership to obtain discounts on publication fees. Need to upgrade your membership level? Please contact Customer Service at Service@asmusa.org.

The ASM Journals program strives for constant improvement in our submission and publication process. Please tell us how we can improve your experience by taking this quick Author Survey.

Thank you for submitting your paper to Spectrum.

Sincerely,
Hyun Jin Kwun
Editor
Microbiology Spectrum

Reviewer #2 (Comments for the Author):

The authors correctly responded to my comments.

Reviewer #3 (Comments for the Author):

The manuscript investigated the effects of specific BLV LTR and tax substitutions on the regulation of viral productivity and transmission by using a longitudinal analysis of BLV sequences and predicted a potential future dominant strain of BLV as the high HVP halotype such as "TTQ".
The reviewers' comments are mostly addressed. Some minor changes/explanations are still needed.

1. Fig. 1D, Figure Caption is not correct or confusing. It should not be the same as 1C.
Parent clones should be LTR 175C (Blue), Mutant clones (Red) should be (LTR175A).

2. Line 96, "the wild-type (WT) thymidine variant (LTR175T)" - please delete "variant". Or change the variant to "strain".

3. Fig. 2, could you add what these arrows indicate in the figure?

4. The strain AN003 (Fig. S5) has a CMR type like AN006, but it does not have the same effect (Fig. 1). Could this be clarified or discussed further? AF547 (TTQ type) vs AJ007 as well? Are there any other factors at play?

5. Fig. 3E is performed in which cell line? It is over expressed with EF1a promoter, not by the viral LTR. Thus it does not necessarily say if each clone has the same level of expression. It could have different stability or be differentially expressed by the LTR175C vs LTR175T or different CREB binding.

6. Line 263, Why is the TTR wild-type? Isn't it TMQ?

We sincerely thank the reviewer for their valuable time and insightful comments on our manuscript. Their constructive feedback has significantly improved the quality of our work. The reviewers' comments have been mostly addressed, and we have made the following changes in response to the remaining minor points.

The manuscript investigated the effects of specific BLV LTR and tax substitutions on the regulation of viral productivity and transmission by using a longitudinal analysis of BLV sequences and predicted a potential future dominant strain of BLV as the high HVP halotype such as "TTQ".

The reviewers' comments are mostly addressed. Some minor changes/explanations are still needed.

1. Fig. 1D, Figure Caption is not correct or confusing. It should not be the same as 1C. Parent clones should be LTR 175C (Blue), Mutant clones (Red) should be (LTR175A).

Answer: We thank the reviewer for pointing this out. The figure caption for Fig. 1D has been revised to clearly distinguish it from Fig. 1C. To avoid any confusion, we have adopted a consistent color scheme throughout both panels: blue bars always indicate parental clones, and red bars always indicate mutant clones. Specifically, in Fig. 1C, the parental clones carry LTR175T (blue) and the mutants carry the T175C substitution (red); in Fig. 1D, the parental clones carry LTR175C (blue) and the mutants carry the C175T substitution (red). (We note that the reviewer's comment referred to "LTR175A," which we interpret as a typographical error for C175T.)

2. Line 96, "the wild-type (WT) thymidine variant (LTR175T)" - please delete "variant". Or change the variant to "strain".

Answer: As suggested, we have revised the text to refer to the "wild-type (WT) thymidine strain (LTR175T)" to improve clarity.

3. Fig. 2, could you add what these arrows indicate in the figure?

Answer: We have added a descriptive note to the Result section (lines 200-202 and 218) and figure legend (lines 757-758) to clarify that the light blue, magenta, and green arrows indicate the positions of residues 73, 69, and 146, respectively, to highlight the localized

structural changes.

4. The strain AN003 (Fig. S5) has a CMR type like AN006, but it does not have the same effect (Fig. 1). Could this be clarified or discussed further? AF547 (TTQ type) vs AJ007 as well? Are there any other factors at play?

Answer: We thank the reviewer for raising this important point. As the reviewer correctly noted, strain AN003 shares the same CMR genotype (LTR175C, Tax69M, Tax73R) with AN006, yet does not exhibit the same low viral productivity (LVP) phenotype. Similarly, strain AF547 possesses the TTQ genotype (LTR175T, Tax69T, Tax73Q) like AJ007, but shows different viral productivity characteristics. These observations strongly suggest that additional genetic variations outside the three loci examined in this study modulate viral productivity in certain strain backgrounds.

To address this issue, we performed whole-genome comparisons between AN003 and AN006, and between AF547 and AJ007. All consensus sequences used for these comparisons (Figs. S2, S5, and S6) were derived from a multiple sequence alignment of 115 BLV strains (Tables S1 and S2), ensuring a robust and consistent reference for variant identification. As shown in newly added Fig. S6, these strains carry distinct nucleotide substitutions across multiple regions of the viral genome. Specifically, AF547 carries not only an LTR mutation (nt 160) but also missense mutations in the gag-pro-pol region (nt 939 and 3605) compared with AJ007. The mutation at nt 939 is located adjacent to the PPPY motif, which regulates viral particle production, whereas the mutation at nt 3605 lies within the polymerase domain, which is critical for viral replication. Similarly, AN003 carries an LTR mutation and a missense mutation in the *G4* gene (nt 7195) that is absent in AN006. Given that G4 is known to regulate viral production, this mutation may functionally compensate for the reduced transactivation associated with the Tax69M and Tax73R substitutions.

We have now incorporated this discussion into both the Results and Discussion sections. In the Results section (lines 261-279), we describe the phenotypic heterogeneity among strains sharing identical LTR and Tax genotypes and present the whole-genome comparison data (Fig. S6). In the Discussion section (lines 358-368 and 402-407), we discuss the possibility that additional genetic variations—including the *gag-pro-pol* and *G4* mutations identified here—may serve as genetic modifiers that influence viral

productivity through compensatory or epistatic interactions. These findings highlight the polygenic nature of BLV viral productivity and underscore the need for comprehensive whole-genome approaches to fully elucidate the genetic determinants of viral fitness. We have also added this perspective to the Discussion to acknowledge the complexity of the genetic regulation of BLV viral productivity.

5. Fig. 3E is performed in which cell line? It is over expressed with EF1a promoter, not by the viral LTR. Thus it does not necessarily say if each clone has the same level of expression. It could have different stability or be differentially expressed by the LTR175C vs LTR175T or different CREB binding.

Answer: We thank the reviewer for this important methodological question.

Cell line: The western blot analysis shown in Fig. 3E was performed using 293T cells, which were transfected with the pBApo-EF1α-Tax-HiBiT expression vectors. We have now clarified this in the Materials and Methods section (line 557) and in the Results section (line 252).

Regarding the EF1α promoter: The reviewer raises an excellent point. We fully acknowledge that the EF1α promoter-driven overexpression system does not replicate the native transcriptional regulation of Tax by the viral LTR. However, the primary purpose of this experiment was to determine whether the observed differences in transactivation activity (Figs. 3C and 3D) were due to alterations in Tax protein expression levels or intracellular stability, rather than to intrinsic functional differences in the mutant Tax proteins. Our results showed that all Tax variants (wild-type and mutants) were expressed at equivalent levels under the constitutive EF1α promoter (Fig. 3E), ruling out the trivial explanation that the transactivation differences were due to variations in Tax protein abundance or stability. This conclusion is valid regardless of the promoter used, as the experiment was designed to compare protein expression levels under identical, controlled conditions.

Regarding LTR175C vs. LTR175T and CREB binding: The reviewer is correct that the EF1α promoter-driven system does not address whether the Tax mutations affect expression levels when driven by the viral LTR, or whether the LTR175C/T genotypes influence Tax expression through differential CREB binding or other transcriptional

mechanisms. We have now added a statement to the Discussion acknowledging this limitation (Discussion, lines 396-402).

We appreciate the reviewer's careful reading and have incorporated these clarifications into the manuscript.

6. Line 263, Why is the TTR wild-type? Isn't it TMQ?

Answer: We thank the reviewer for this question. The designation of T-T-R (LTR175T, Tax69T, Tax73R) as the wild-type haplotype was based on the consensus sequence generated from the alignment of 115 BLV strains analyzed in this study (Tables S1 and S2). At each of the three loci examined, the most frequently observed nucleotide or amino acid residue was defined as the consensus:

- LTR175: Thymidine (T) was the most frequent nucleotide
- Tax69: Threonine (T) was the most frequent amino acid
- Tax73: Arginine (R) was the most frequent amino acid

Therefore, the T-T-R haplotype represents the majority-rule consensus and was designated as the wild-type, with the other haplotypes (C-T-R, T-T-Q, and T-M-R) representing variants. We have now clarified this in the manuscript (Results, lines 288-291): "The T-T-R haplotype was considered the wild-type, based on the consensus sequence derived from the majority-rule alignment of 115 BLV strains (Tables S1 and S2; the nucleotide and amino acid frequencies at the three loci are summarized in Fig. S5), with the others representing variants."

We hope this clarifies our rationale for designating T-T-R as the wild-type haplotype.

Re: Spectrum00869-26R2 (**Combinations of long terminal repeat and Tax protein substitutions influence bovine leukemia virus transmission through altered viral productivity**)

Dear Dr. Hironobu Murakami:

Your manuscript has been accepted, and I am forwarding it to the ASM production staff for publication. Your paper will first be checked to make sure all elements meet the technical requirements. Please contact ASM staff to submit the revised figure before copyediting and production can begin.

Data Availability: ASM policy requires that data be available to the public upon online posting of the article, so please verify all links to sequence records, if present, and make sure that each number retrieves the full record of the data. If a new accession number is not linked or a link is broken, provide production staff with the correct URL for the record. If the accession numbers for new data are not publicly accessible before the expected online posting of the article, publication may be delayed; please contact ASM production staff immediately with the expected release date.

Publication Fees: For information on publication fees and which article types have charges, please visit our website. We have partnered with Copyright Clearance Center (CCC) to collect author charges. If fees apply to your paper, you will receive a message from no-reply@copyright.com with further instructions. For questions related to paying charges through RightsLink, please contact CCC at ASM_Support@copyright.com or toll free at +1-877-622-5543. CCC makes every attempt to respond to all emails within 24 hours.

ASM Membership: Corresponding authors may join or renew ASM membership to obtain discounts on publication fees. Need to upgrade your membership level? Please contact Customer Service at Service@asmusa.org.

Embargo Policy: A press release may be issued as soon as the manuscript is posted on the Spectrum Latest Articles webpage. The corresponding author will receive an email with the subject line "ASM Journals Author Services Notification" when the article is available online.

The ASM Journals program strives for constant improvement in our submission and publication process. Please tell us how we can improve your experience by taking this quick Author Survey.

Thank you for submitting your paper to Spectrum.

Sincerely,
Hyun Jin Kwun
Editor
Microbiology Spectrum
