# Supplementary material for: Combinations of long terminal repeat and tax protein substitutions influence bovine leukemia virus transmission through altered viral productivity
Source: Microbiol Spectr. 2026 Jul 27;14(9):e00869-26. doi: 10.1128/spectrum.00869-26 (PMC13532317; doi:10.1128/spectrum.00869-26)
Supplement: Supplementary file 2 [file spectrum.00869-26-s0001.pdf]

Supplementary Tables

Table S1. Strains cloned in this study.

Strain	Accession number
AF028	LC760082
AF052	LC760083
AF073	LC760084
AF074	LC760085
AF157	LC760086
AF173	LC760087
AF193	LC760088
AF208	LC760089
AF227	LC760090
AF234	LC760091
AF241	LC760092
AF250	LC760093
AF265	LC760094
AF293	LC760095
AF469	LC760096
AF540	LC760097
AF547	LC760098
AF557	LC760099
AF564	LC760100
AF604	LC760101
AF605	LC760102
AF629	LC760103
AF642	LC760104
AF658	LC760105
AF659	LC760106
AF746	LC760107
AF784	LC760108
AF800	LC760109
AF893	LC760110
AH001	LC760111
AH002	LC760112
AH003	LC760113
AH004	LC760114
AH005	LC760115
AH006	LC760116
AH007	LC760117
AK002	LC760118
AK004	LC760119
AK008	LC760120
AK012	LC760121
AK014	LC760122
AK015	LC760123
AK016	LC760124
AK017	LC760125
AK018	LC760126
AK021	LC760127
AK022	LC760128
AK023	LC760129
AK024	LC760130
AS003	LC760131
AS005	LC760132
AS008	LC760133
AS010	LC760134
AS011	LC760135
AS012	LC760136
AS015	LC760137

Table S2. Previously reported strains used in this study.

Strain	Accession number
AF060	AP018006
AF076	AP018007
AF245	AP018009
AF266	AP018010
AF438	AP018012
AF481	AP018013
AF513	AP018014
AF805	AP018017
AF902	AP018018
AF967	LC164085
AF982	AP018019
AJ001	AP019565
AJ002	AP019566
AJ003	AP019567
AJ004	AP019568
AJ005	AP019569
AJ006	AP019570
AJ007	AP019571
AJ008	AP019572
AJ009	AP019573
AJ010	AP019574
AJ011	AP019575
AJ012	AP019576
AJ013	AP019577
AJ014	AP019578
AJ015	AP019579
AJ016	AP019580
AJ017	AP019581
AJ018	AP019582
AJ019	AP019583
AJ020	AP019584
AJ021	AP019585
AJ022	AP019586
AJ023	AP019587
AJ024	AP019588
AJ025	AP019589
AJ026	AP019590
AJ027	AP019591
AJ028	AP019592
AJ029	AP019593
AJ030	AP019594
AJ031	AP019595
AJ032	AP019596
AJ033	AP019597
AJ034	AP019598
AK001	AP018020
AK006	AP018021
AK007	AP018022
AK011	AP018023
AN003	AP018024
AN004	AP018025
AN006	AP018026
AN008	AP018027
AN009	AP018028
AN011	AP018029
AN013	AP018030
AN014	AP018031
AN015	AP018032
AN903	LC164086

Table S3. Structural superposition analysis of Tax protein models.

Parameter	Consensus vs. AJ007	Consensus vs. AN006
Sequence score	1488.6	1488.4
RMSD (pruned)	1.291 Å	1.318 Å
Pruned pairs	39/309	56/309
RMSD (all)	16.556 Å	11.199 Å

Table S4. Pairwise comparisons of PVL among haplotypes.

Comparison	Estimate	SE	t value	P value
C-T-R vs T-M-R	-1.017	0.918	-1.108	0.686
C-T-R vs T-T-Q	-0.485	0.299	-1.621	0.371
C-T-R vs T-T-R	-0.117	0.235	-0.5	0.959
T-M-R vs T-T-Q	0.531	0.955	0.556	0.945
T-M-R vs T-T-R	0.899	0.935	0.962	0.771
T-T-Q vs T-T-R	0.368	0.354	1.04	0.726

Table S5. Primers used for SNP genotyping.

Primer	Sequence (5'-3')	Detection
Allele Specific Primer 1	GACAGGGCCGGCATTTTATTAA	LTR175T/C
Allele Specific Primer 2	GACAGGGCCGGCATTTTATTAG	
Locus Specific Primer	GCCGTACCTCCCCAACTTCC	
Allele Specific Primer 1	TTGCGGCCTCAATGGAAC	Tax69T/M
Allele Specific Primer 2	CTTGCGGCCTCAATGGAAT	
Locus Specific Primer	GCTGGCATTGGTAGGGCTG	
Allele Specific Primer 1	TCTCGGAGACATGCAGTC	Tax73R/Q
Allele Specific Primer 2	TCTCGGAGACATGCAGTT	
Locus Specific Primer	GCGCCCTGGTTTTGTCCAATG	

Table S6. Primer pairs for vector construction.

Primer pair	Sequence (5'–3')
ΔRLuc	TTCTAGAGCGGCCGCTTCGA
	GGTGGCTAGCCTATAGTGAG
FLuc	TATAGGCTAGCCACCATGGAAGACGCCAAAAACAT
	GCGGCCGCTCTAGAATTACACGGCGATCTTTCCG
ΔTK	AAGCTTGATTCTTCTGACACAACAGTCTCG
	TAGATCTGTCGAGCCATGTGAGCAAAAGGC
U3	GGCTCGACAGATCTATGTATGAAAGATCATGCCGACCTA
	AGAAGAATCAAGCTTTGGTGCCGCTAACTCGACAGGGCCG
pBA	CGGTGTGAGCGGCTGGCGGCTGTTCAAGAAGATTAGCTGACATGCAAGCTTGGGGGAGGCTAACT
	AGGTCGACTCTAGAGGATCC
Tax	CAGCCGCTCACACCGCTCGAGCCGCCAGAACTCCCAAAAAGGCGGGAGAGCCATTCATTT
	CTCTAGAGTCGACCTATGGCAAGTGTTGTTGGTTGGGGGCCCCACTCTC

Table S7. Primers used for point mutation induction.

Primer	Sequence (5'-3')	Induction of mutation
TAX69M-F	CCTCAATGGAATGTTGTTCCCTC	T69M
TAX69M-R	AACATTCCATTGAGGCCGCAAGGTC	
TAX69T-F	CCTCAATGGAACGTTGTTCCCTC	M69T
TAX69T-R	AACGTTCCATTGAGGCCGCAAGGTC	
TAX73R-F	TCCCTCGACTGCATGTCTCCGA	T73R
TAX73R-R	CATGCAGTCGAGGGAACAAC	
TAX73Q-F	TCCCTCAACTGCATGTCTCCGA	R73Q
TAX73Q-R	CATGCAGTTGAGGGAACAAC	

Supplementary Figures

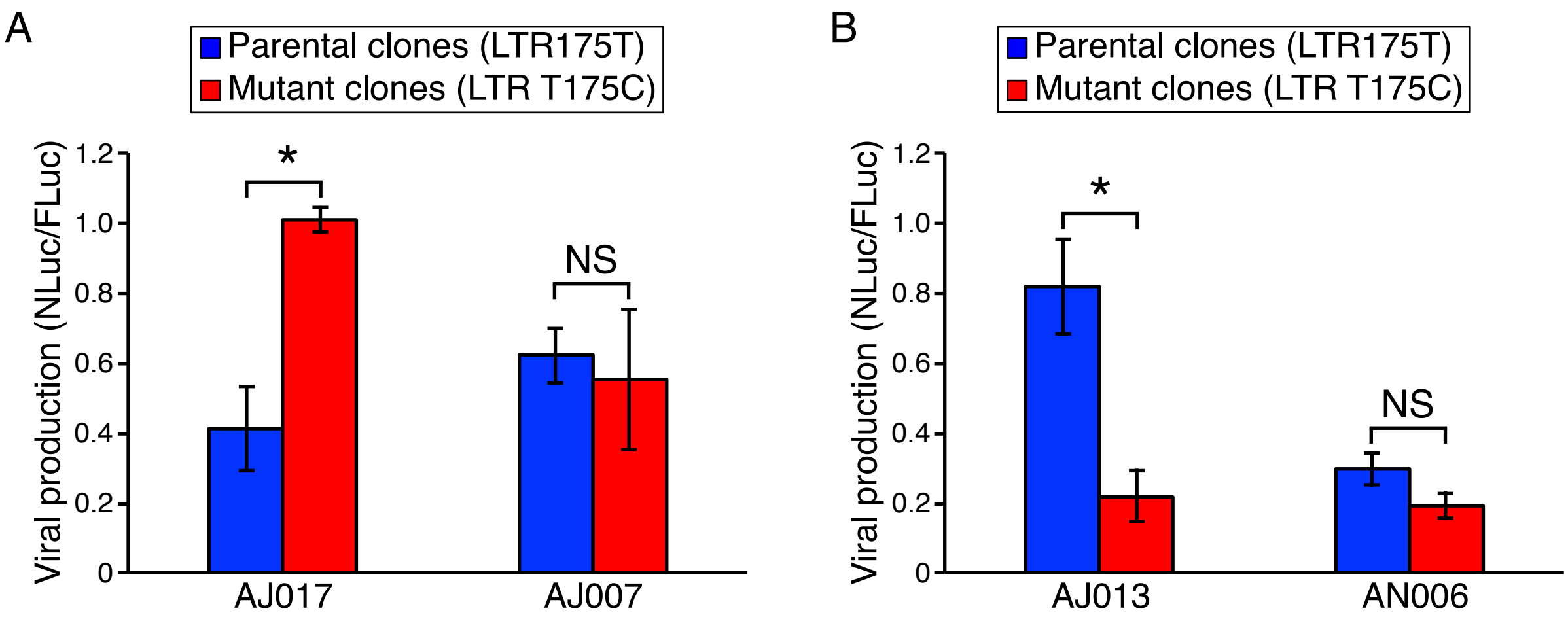

Fig. S1. Validation of LTR175 substitution effects in bovine MAC-T cells. (A, B) Representative and specific molecular clones were evaluated in bovine epithelial cells. Point mutations were introduced: T175C in LTR175T parental strains (A) and C175T in LTR175C parental strains (B). Blue and red bars indicate parental and mutant clones, respectively. **p* < 0.05. NS: no significant difference (*p* > 0.05, Student's *t*-test).

Region	5' LTR																	AS2																					
					gag-pro-pol																																		
Position	74	175	528	573	730	858	890	953	954	1190	1313	1346	1499	1514	1596	1790	1849	2032	2233	2314	2404	2595	2763	2844	3021	3108	3219	3318	3453	3565	3605	3612	3653	3693	3741	3843	3852	3924	4182	4558
Consensus	A	T	A	C	C	T	A	T	T	T	G	A	T	A	A	A	C	G	C	T	T	T	C	G	T	T	T	C	T	T	C	T	C	A	A	A	T	T	C	T
AJ007	G	.	.	T	.	C	T	.	C	A	A	G	C	G	.	.	T	A	.	C	C	.	T	A	.	C	.	T	C	.	A	.	.	.	.	.	C	.	T	.
AN006	.	C	G	.	T	.	.	C	.	.	.	.	.	.	G	C	.	.	T	.	.	C	.	.	C	.	C	.	.	C	.	C	A	G	G	G	.	C	.	G

Region													AS1	rex					3' LTR	
	env													tax						
Position	4909	4976	5076	5392	5571	5722	5877	5949	5979	6031	6256	6466	7012	7475	7487	7914	7989	8016	8285	8386
Consensus	C	C	A	A	C	G	T	A	T	A	A	A	G	C	G	C	C	T	A	C
AJ007	G	T	.	C	T	A	A	G	C	G	C	.	A	.	A	T	T	C	G	T
AN006	.	.	G	.	.	.	.	.	.	.	.	G	.	T	.	.	.	.	.	.

Fig. S2. Summary of nucleotide and amino acid substitutions in AJ007 and AN006 strains. Numbers indicate coding gene locations; dots indicate residues identical to the consensus sequence.

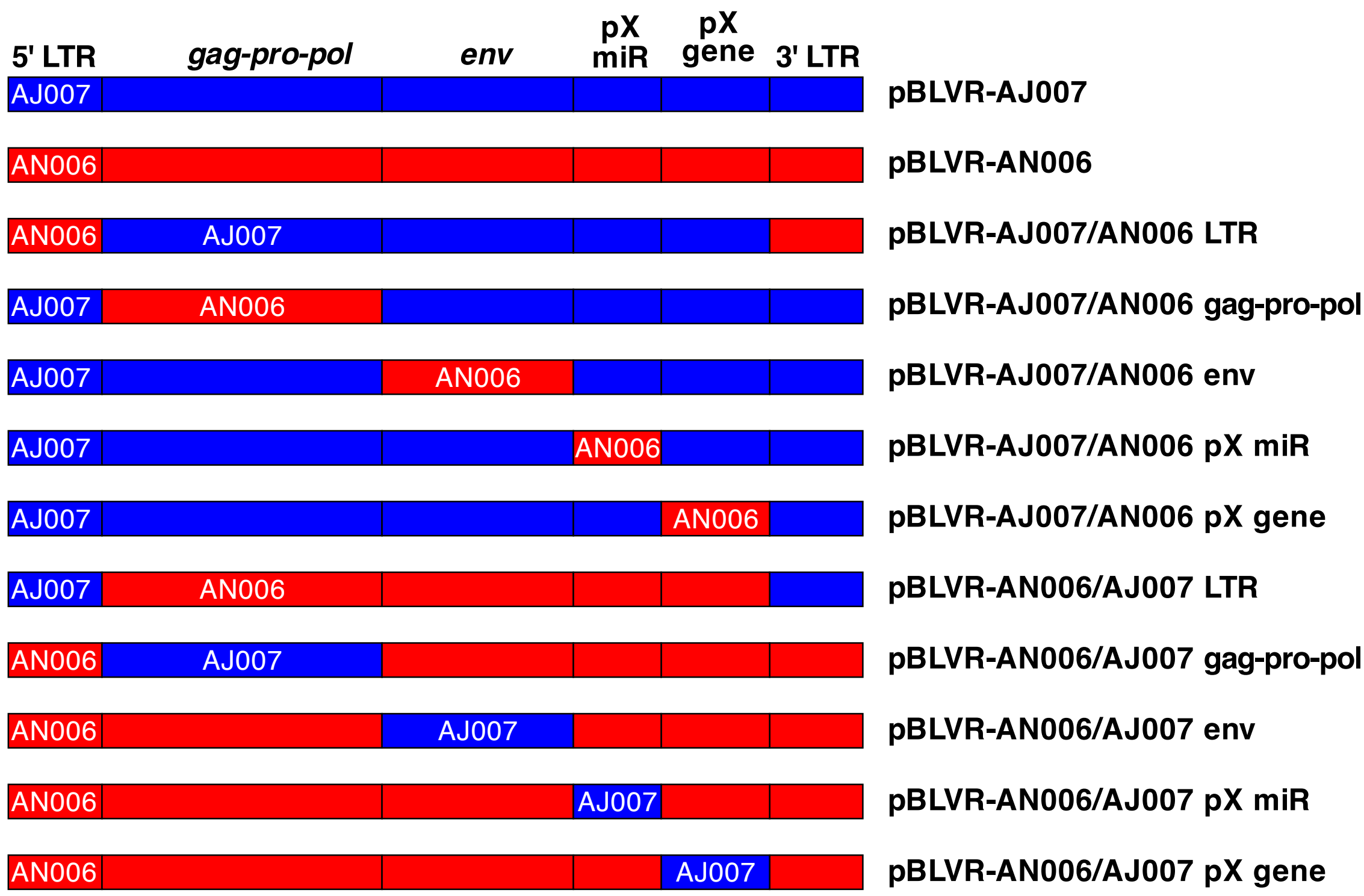

Fig. S3. Schematic diagrams of wild-type and chimeric viral whole-genomes. Left and right sides represent 5′ and 3′ termini. Blue and red bars indicate pBLVR-AJ007 and -AN006 sequences, respectively.

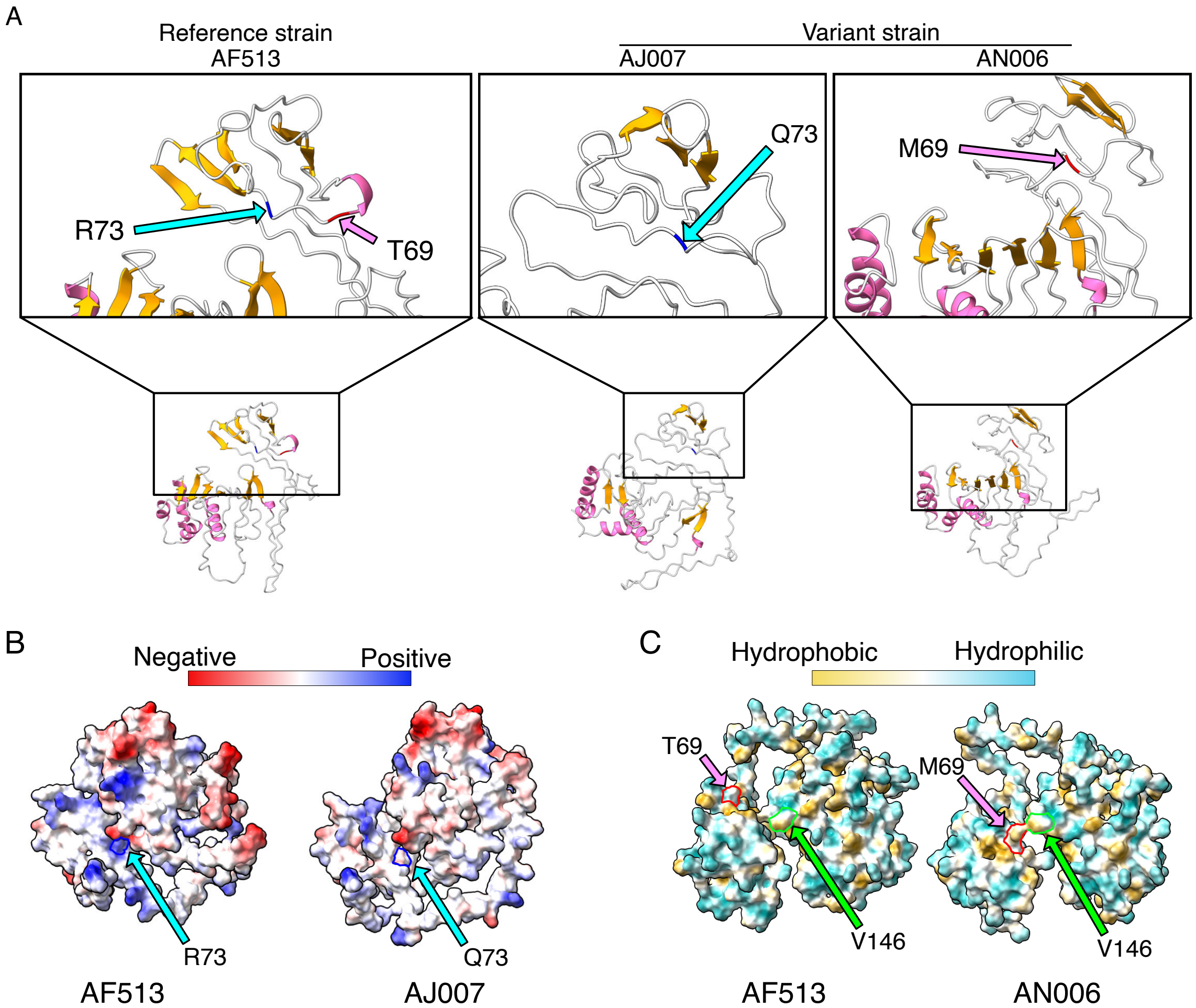

Fig. S4. Detailed ribbon models and local amino acid topology of AlphaFold2-predicted Tax structures. (A) Ribbon-based structural superposition of the Tax proteins from the AF513 reference strain (left), the AJ007 variant (center), and the AN006 variant (right). Predicted Tax protein structures are indicated as follows: pink, α-helices; orange, β-strands; and white, random coils. Magenta and light blue arrows indicate the substitution sites at positions 69 and 73, respectively. The top insets present a close-up view of the peripheral loop regions containing amino acid residues 69 and 73. (B) Electrostatic potential maps of Tax reference and variant proteins. Positively and negatively charged regions are shown in blue and red, respectively, with neutral surfaces shown in white. The residue at position 73 is outlined in blue. (C) Surface hydrophobicity of Tax proteins. Tan,

hydrophobic areas; cyan, hydrophilic areas. Residues 69 and 146 are indicated by magenta and green arrows, respectively (also outlined in red and green).

Region	LTR	Tax	
Location	nt 175	aa 69	aa 73
Consensus	T	T	R
AF245	.	.	Q
AF547	.	.	Q
AF982	.	.	Q
AJ007	.	.	Q
AJ017	.	.	Q
AJ030	.	.	Q
AN003	C	M	.
AN006	C	M	.
AN009	C	M	.

Fig. S5. Substitutions at LTR175, Tax69, and Tax73. Dots indicate residues identical to the consensus sequence. The consensus residue at each locus was defined as the most frequently observed nucleotide or amino acid among the 115 strains. The T-T-R haplotype (LTR175T, Tax69T, Tax73R) represents this consensus.

A

Region			AS2	
	5' LTR	gag-pro-pol		
Position	160	939	2404	3605
Consensus	T	G	T	C
AF547	C	A	.	.
AJ007	.	.	C	A

B

Region						AS2					G4			
	5' LTR	gag-pro-pol							env		AS1	tax		3' LTR
Position	252	951	953	1581	1790	2765	3565	3612	4904	6345	7195	7809	8106	8463
Consensus	T	G	T	A	A	A	T	T	T	C	A	A	C	T
AN003	C	.	.	G	.	G	.	.	C	T	C	G	A	C
AN006	.	A	C	.	C	.	C	C	.	.	.	.	.	.

Fig. S6. Nucleotide sequence comparison of BLV strains. (A) Nucleotide substitutions between AF547 and AJ007. (B) Nucleotide substitutions between AN003 and AN006. Nucleotide positions are numbered according to their respective genomic locations. Dots indicate residues identical to the consensus sequence, which was derived from the 115 BLV strains analyzed in this study.

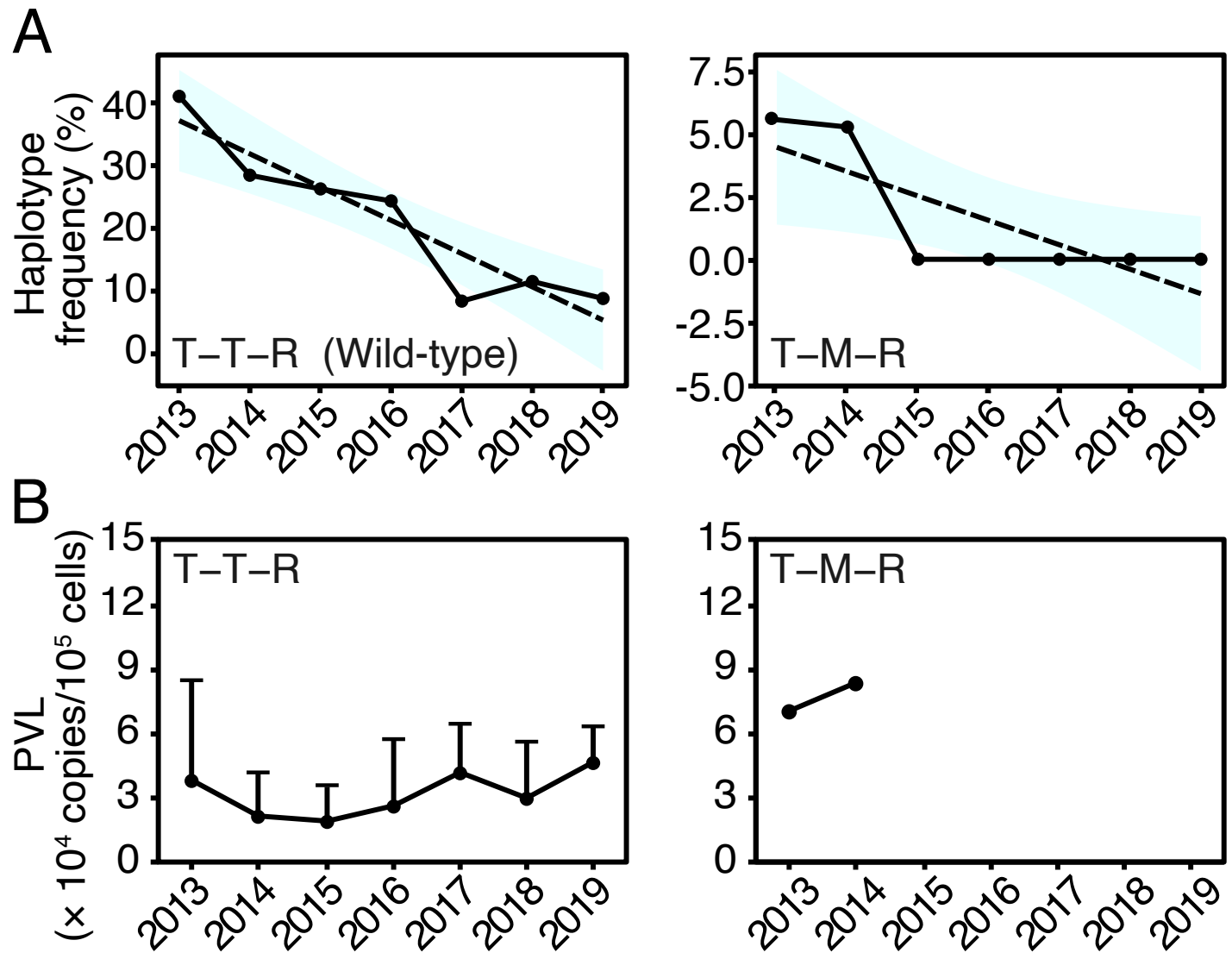

Fig. S7. Longitudinal BLV strain frequencies and PVL in LVP haplotypes. (A) Annual relative frequency of LVP haplotypes. Solid black lines represent observed temporal changes; dashed black lines indicate linear regression trends, with blue-shaded areas denoting the 95% confidence intervals of regression. (B) PVL among HVP haplotypes plotted as means ± standard deviations per year.
